# Depletion of tumor associated macrophages enhances local and systemic platelet-mediated anti-PD-1 delivery for post-surgery tumor recurrence treatment

**DOI:** 10.1038/s41467-022-29388-0

**Published:** 2022-04-06

**Authors:** Zhaoting Li, Yingyue Ding, Jun Liu, Jianxin Wang, Fanyi Mo, Yixin Wang, Ting-Jing Chen-Mayfield, Paul M. Sondel, Seungpyo Hong, Quanyin Hu

**Affiliations:** 1 Pharmaceutical Sciences Division, School of Pharmacy, University of Wisconsin-Madison, Madison, WI 53705 USA; 2 Carbone Cancer Center, School of Medicine and Public Health, University of Wisconsin-Madison, Madison, WI 53705 USA; 3 Wisconsin Center for NanoBioSystems, School of Pharmacy, University of Wisconsin-Madison, Madison, WI 53705 USA; 4 Department of Human Oncology, University of Wisconsin School of Medicine and Public Health, Madison, WI USA; 5 Department of Pediatrics, University of Wisconsin School of Medicine and Public Health, Madison, WI USA

**Keywords:** Drug delivery, Cancer immunotherapy, Tumour immunology

## Abstract

Immunosuppressive cells residing in the tumor microenvironment, especially tumor associated macrophages (TAMs), hinder the infiltration and activation of T cells, limiting the anti-cancer outcomes of immune checkpoint blockade. Here, we report a biocompatible alginate-based hydrogel loaded with Pexidartinib (PLX)-encapsulated nanoparticles that gradually release PLX at the tumor site to block colony-stimulating factor 1 receptors (CSF1R) for depleting TAMs. The controlled TAM depletion creates a favorable milieu for facilitating local and systemic delivery of anti-programmed cell death protein 1 (aPD-1) antibody-conjugated platelets to inhibit post-surgery tumor recurrence. The tumor immunosuppressive microenvironment is also reprogrammed by TAM elimination, further promoting the infiltration of T cells into tumor tissues. Moreover, the inflammatory environment after surgery could trigger the activation of platelets to facilitate the release of aPD-1 accompanied with platelet-derived microparticles binding to PD-1 receptors for re-activating T cells. All these results collectively indicate that the immunotherapeutic efficacy against tumor recurrence of both local and systemic administration of aPD-1 antibody-conjugated platelets could be strengthened by local depletion of TAMs through the hydrogel reservoir.

## Abstract (web summary)

Increased density of tumor associated macrophages has been correlated with tumor recurrence following surgery. Here the authors design an alginate-based hydrogel encapsulating anti-PD-1-conjugated platelets and nanoparticles loaded with the macrophage-depleting CSF-1R inhibitor pexidartinib, showing inhibition of post-surgery tumor recurrence in preclinical models.

## Introduction

Surgery remains the foremost treatment option for patients with solid tumors, while tumor recurrence frequently occurs, leading to a low rate of long-term survival^1,2^. Cancer immunotherapy, especially immune checkpoint blockade, has been demonstrated as one of the most potent anti-cancer recurrence strategies either as monotherapies or in combinations with other treatment modalities^3–7^. Specifically, inhibition of binding between programmed cell death protein 1 (PD-1) and programmed cell death ligand 1 (PD-L1) has been demonstrated to regain suppressed anti-tumor immunity, which has great potential to effectively treat cancer recurrence in the clinical settings^8,9^. However, intricate physiological environment changes after surgery, especially wound healing-triggered inflammatory condition and inflammation-responsive immunosuppression, could diminish the efficacy of cancer immunotherapy, leading to the low objective response rate in the clinic^10–12^. It is worth noting that a large number of inflammatory cells represented by macrophages participate in the process of surgical wound healing and tissue repair. Moreover, tumor-associated macrophages, especially M2 type macrophages, secrete a large amount of anti-inflammatory factors, such as IL-10, to form an immunosuppressive microenvironment, thereby helping the remaining tumor cells escape immune surveillance, and accelerate their recurrence and metastasis. Furthermore, immune-related adverse events due to over-activated T cells by immune checkpoint blockade remain a concern^13,14^.

The connection between TAMs and cancer recurrence after surgery has been established in both preclinical studies and clinical settings. It has been demonstrated that surgery could enhance the proliferation of tumor cells locally and in distant metastases, which is correlated with the increased macrophage density in the tumor tissues after surgery^15^. Additionally, the depletion of TAMs resulted in inhibited tumor growth after surgery. For example, a single-cell protein activity analysis was applied to identify one specific macrophage population that correlated positively with clear cell renal carcinoma (ccRCC) recurrence after surgery^16^, which has also been validated in a large clinical validation cohort, in which this sub-population of macrophages were significantly enriched in tumors from patients who experienced tumor recurrence following surgery. Furthermore, in clinical settings, TAMs have been frequently applied as prognostic markers for multiple cancer recurrences after surgery including non-small cell lung cancer, gastric cancer, and non-functional pancreatic neuroendocrine tumor^17–19^. Collectively, all these pre-clinical and clinical studies validate that there is a strong correlation between enriched TAMs and tumor recurrence after surgery, and TAM depletion could have a positive impact on preventing post-surgical tumor recurrence.

One more mechanism that limits the immune checkpoint blockade therapy in recurrent cancer is the hindered T cell migration towards and within the tumor site due to tumor immunosuppressive microenvironment, resulting in the low availability of tumor infiltrating lymphocytes and progressive tumor growth^20,21^. Studies have shown that immunosuppressive cells, especially TAMs, are highly implicated in suppressing anti-tumor immune functions of T cells and directly facilitate tumor cell immune escape^22,23^. Specifically, a recent study has identified that CD8^+^ T cell accumulation and infiltration at tumor sites have been primarily enhanced by depleting TAMs, resulting in improved immunotherapy efficacy^24^. Pexidartinib is an FDA-approved small molecule drug that shows remarkable selectivity to block the CSF1 receptors on TAMs with negligible cytotoxicity against normal cells, favoring itself as a potential target for TAM depletion for modulating the tumor immunosuppressive microenvironment and enhancing immunotherapy by improving T cell infiltration^25,26^. Thus, the combination of TAM depletion and immune checkpoint blockade could be critical for augmenting anti-tumor immunotherapy outcomes.

**Fig. 1:**
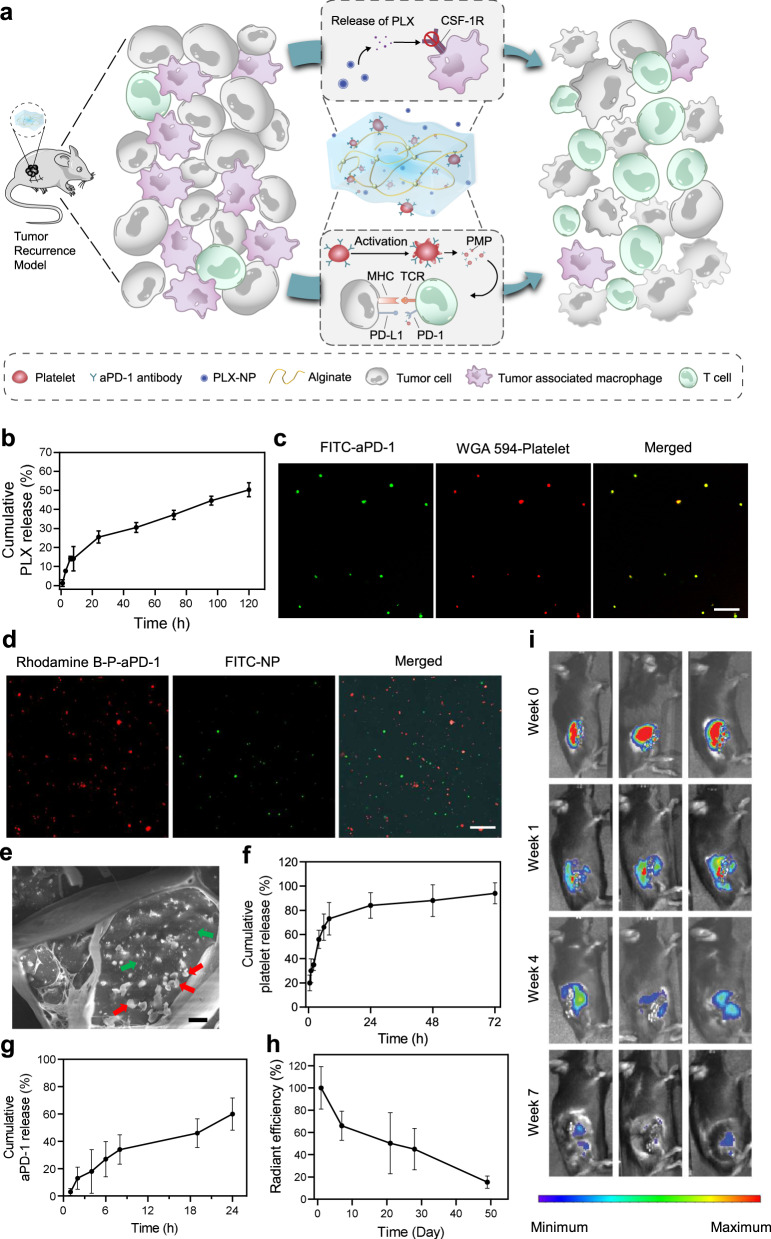
Preparation and characterization of PLX-NP-P-aPD-1@Gel. **a** Schematic illustration of the mechanism of tumor immune suppressive microenvironment modulation capability of PLX-NP and P-aPD-1 loaded alginate-based hydrogel in the tumor recurrence model. MHC, major histocompatibility complex; TCR, T-cell receptor. **b** Release profile of PLX-NP in vitro at pH of 6.5. Data are presented as mean ± s.d. (*n* = 3). **c** Confocal microscopy images of anti-PD-1-conjugated platelets. Scale bar, 20 µm. Green: FITC-labeled aPD-1; Red: WGA 594-labeled platelet. The experiments were repeated three times. **d** Confocal microscopy images of NP and P-aPD-1 in the hydrogel. Scale bar, 50 µm. Green: FITC-labeled NP; Red: Rhodamine B-labeled P-aPD-1. The experiments were repeated three times. **e** The Cryo SEM image of PLX-NP and P-aPD-1 co-loaded hydrogel. (Scale bar = 2 µm, red arrow: platelets, green arrow: PLX-NP). The experiments were repeated three times. **f**, **g** The in vitro release profiles of platelets (**f**) and aPD-1 (**g**) from the hydrogel. Data are presented as mean ± s.d. (*n* = 3). **h**, **i** Degradation profile of Cy5.5-alginate hydrogel in vivo represented by radiant efficiency (**h**) and IVIS spectrum imaging (**i**). Data are presented as mean ± s.d. (*n* = 3 mice). Source data are provided as a Source Data file.

Here, we show a biocompatible alginate-based hydrogel encapsulating PLX-loaded nanoparticles (designated PLX-NP) and anti-PD-1-conjugated platelets (designated P-aPD-1) for post-surgery tumor recurrence treatment, to achieve local and sustained release of PLX and anti-PD-1 antibodies for depleting TAMs and re-activating infiltrated T cells, respectively (Fig. 1a). A biodegradable dextran nanoparticle is formulated to encapsulate and bioresponsively release PLX to block CSF1R to eliminate TAMs in the tumor microenvironment. Anti-PD-1 antibodies are conjugated on the surface of platelets, where the inflammatory environment secondary to the surgical exposure could activate platelets to release anti-PD-1 antibodies to block PD-1 receptors on infiltrated T cells in the format of PMP-aPD-1 (platelet-derived microparticles with anti-PD-1 antibodies)^27,28^. Both PLX-NP and P-aPD-1 are harbored in the hydrogel to form a local delivery reservoir, in which PLX is gradually released to deplete TAMs to recruit T cells toward tumor parenchyma, favoring the subsequent efficacy of the anti-PD-1 immunotherapy. To further extend the application of this combination immunotherapy strategy, P-aPD-1 is systemically administered to synergize with local PLX-NP-loaded hydrogel to promote a sustained immune response against tumor recurrence, where less than 20% of patients are benefiting from a systemic injection of checkpoint inhibitors clinically^29^. Taken together, this hydrogel-based delivery system could enhance post-surgery tumor recurrence treatment by gradually releasing PLX to deplete TAMs, facilitating T cell recruitment and infiltration, and promoting both local and systemic platelet-mediated immune checkpoint inhibitor delivery.

## Results

### Preparation and characterization of PLX-NP-P-aPD-1@Gel

PLX-loaded dextran nanoparticles were prepared by a single-emulsion method. The average size of the PLX-NP was determined to be 145.0 nm by the dynamic light scattering (DLS) (Supplementary Fig. 1). Additionally, a representative transmission electron microscopy (TEM) image of the PLX-NP showed the monodispersed PLX-NP with spherical morphologies (Supplementary Fig. 1). The in vitro release of PLX from PLX-NP was then investigated at pH 6.5 to mimic the pH in the acidic tumor microenvironment, and that may well also represent the pH in the residual tumor remaining at the surgical margin following incomplete tumor resection^30–32^. As shown in Fig. 1b, PLX-NP displayed a sustained release of PLX, and the cumulative release percentage reached 50.3% by day 5. Moreover, to study the in vitro macrophage cytotoxicity of PLX-NP, a cellular MTT assay was performed on RAW264.7 cells showing that PLX-NP displayed dose-dependent macrophage-specific cytotoxicity, while having no significant impact on the viability of NIH/3T3 fibroblasts (Supplementary Fig. 2).

Anti-PD-1 antibody-conjugated platelets were prepared by covalently coupling the amine groups on aPD-1 antibodies with the thiol groups on the surface of platelets using Sulfo-SMCC linkers. The successful conjugation was determined using a confocal microscope, as evidenced by the overlap between WGA 594-labeled platelets and fluorescein isothiocyanate (FITC)-labeled aPD-1 antibodies (Fig. 1c). The conjugated amount of aPD-1 was set as 0.1 pg/platelet according to our previous study^33^, which showed negligible cytotoxicity against platelets. To further affirm that the aPD-1 conjugation would not affect the bio-functionality of platelets, a collagen binding assay was performed. As shown in Supplementary Fig. 3, the collagen binding ability of P-aPD-1 was not altered after aPD-1 decoration, compared with naïve platelets. Furthermore, the expression of platelet surface markers, including CD61, CD41, CD9, and CD62P, did not change significantly compared with naïve platelets (Supplementary Fig. 4), indicating that P-aPD-1 reserved intrinsic properties of platelets.

Then, we loaded PLX-NP and P-aPD-1 into a biocompatible alginate hydrogel. As shown in Supplementary Fig. 5, the alginate solution underwent a quick sol-to-gel transition after the addition of Ca^2+^ solutions. To investigate the loading efficiency of PLX-NP and P-aPD-1 in the hydrogel, various amounts of Ca^2+^ solution containing PLX-NP and P-aPD-1 were applied to form alginate hydrogels. 81.0% and 77.9% loading efficiency of P-aPD-1 and PLX-NP were achieved, respectively, when the ratio of Ca^2+^ solution containing P-aPD-1 and PLX-NP, and alginate solution was set as 1:3.3 (Supplementary Fig. 6). The co-existence of PLX-NP and P-aPD-1 in the hydrogel was visualized by confocal imaging and cryo scanning electron microscope (SEM), where both PLX-NP and P-aPD-1 were embedded in a networking structural hydrogel (Fig. 1d, e, Supplementary Fig. 7). Next, the release profiles of both platelets and aPD-1 antibodies from the hydrogel were characterized in transwell devices. As shown in Fig. 1f, g, sustained-release patterns were observed for both platelets and aPD-1 antibodies, in which 84% of platelets and 60% of aPD-1 antibodies were released within 24 h. Also, the PLX was gradually released from the hydrogel-based delivery system in vivo, and ~33.7% of PLX was released to the surgical bed after 5 days (Supplementary Fig. 8). To evaluate the in vivo degradation of the hydrogel, the C57BL/6 mice were surgically implanted with Cy5.5-labeled alginate hydrogel. The hydrogel displayed good biocompatibility and biodegradability in vivo, as evidenced by a decayed fluorescent signal during the time-course of implantation (Fig. 1h, i).

### In vivo TAM depletion capability of PLX-NP-loaded hydrogel

**Fig. 2:**
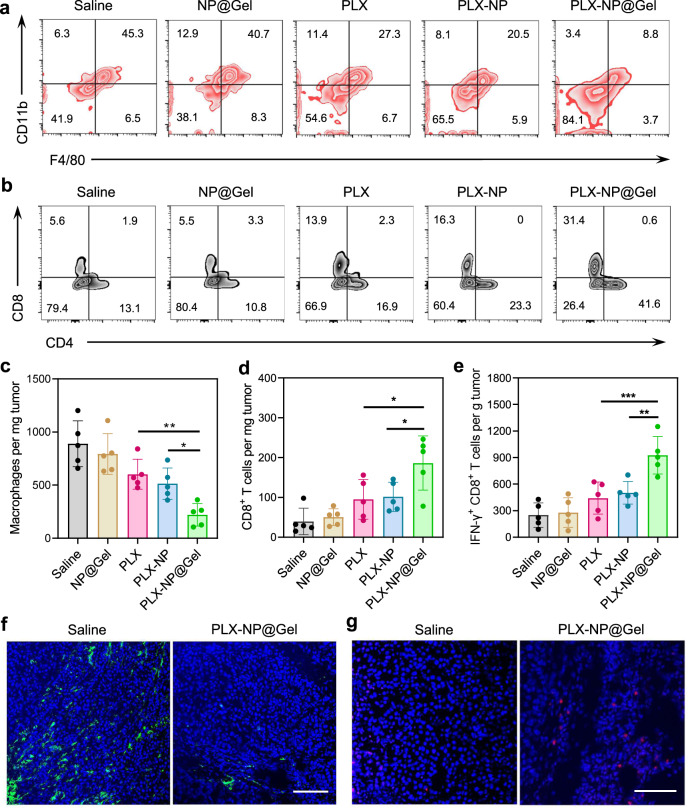
Evaluation of in vivo TAM depletion and T cell infiltration after PLX-NP@Gel treatment in recurrent B16F10 tumor model. **a**, **b** Representative flow cytometry plots of TAMs (**a**) and CD8^+^ T cells (**b**) in the recurrent tumor tissues after treatments with saline, NP@Gel, PLX, PLX-NP, and PLX-NP@Gel. **c**–**e** Quantitative analysis of intratumoral densities of F4/80^+^ macrophages (**c**, PLX-NP@Gel vs. PLX-NP: ^*^P = 0.0358; PLX-NP@Gel vs. PLX: ^**^P = 0.0055), CD8^+^ T cells (**d**, PLX-NP@Gel vs. PLX-NP: ^*^P = 0.0242; PLX-NP@Gel vs. PLX: ^*^P = 0.0147), and IFN*γ*^+^ CD8^+^ T cells (**e**, PLX-NP@Gel vs. PLX-NP: ^**^P = 0.0026; PLX-NP@Gel vs. PLX: ^***^P = 0.0007) per tumor mass in the recurrent tumor tissues after treatments with saline, NP@Gel, PLX, PLX-NP, and PLX-NP@Gel. Data are presented as mean ± s.d. (*n* = 5 biologically independent samples) and analyzed with one-way ANOVA followed by Dunnett’s multiple comparison test. **f**, **g** Representative confocal microscopy images of immune-stained TAMs (**f**) and CD8^+^ T cells (**g**) in the saline group and PLX-NP@Gel group (*n* = 3 biologically independent samples). Scale bar, 100 µm. The experiments were repeated three times. Source data are provided as a Source Data file.

We investigated whether PLX-NP@Gel implanted at the tumor surgical cavity has the ability to deplete TAMs and enhance CD8^+^ T cell infiltration. The melanoma recurrence mouse model was built by surgery, once the tumor size reached about 150 mm^3^. The tumor mass was removed as much as possible during the surgery under a microscope. Afterward, the mice were treated with saline, blank nanoparticle-loaded hydrogel (NP@Gel), free PLX, PLX-NP, PLX-NP-loaded hydrogel (PLX-NP@Gel). As shown in Fig. 2a, the percentages of macrophages in the tumor tissues treated with all PLX formulations were lowered compared with saline and NP@Gel groups, demonstrating the TAM depletion capability of PLX by blocking CSF1R. While there was no substantial difference in TAMs between PLX and PLX-NP groups, which could be attributed to the quick clearance of both free PLX and PLX-NP^34,35^. Furthermore, PLX-NP@Gel displayed a significantly enhanced potency compared to free PLX and PLX-NP groups, as evidenced by 2.3-fold and 1.8-fold decreases in the density of TAMs, highlighting the superiority of hydrogel as a local reservoir for controlled release of PLX-NP for enhanced efficacy (Fig. 2c). In addition, the increased infiltration of CD8^+^ T cells was observed as a result of enhanced TAM depletion. As shown in Fig. 2b, d and Supplementary Fig. 9, PLX-NP@Gel treatment induced 1.8-fold and 2.0-fold increases in CD8^+^ T cell density compared to PLX-NP and PLX groups. Moreover, the density of IFNγ^+^ CD8^+^ T cells in PLX-NP@Gel group was 1.8-fold and 2.1-fold higher than that in PLX-NP and PLX groups (Fig. 2e and Supplementary Fig. 10). To directly visualize the TAM depletion and CD8^+^ T cells infiltration, the tumor tissues were collected and sectioned for immunostaining. A substantial decrease of TAMs and an increase of infiltrated CD8^+^ T cells were observed in confocal images in comparison of saline and PLX-NP@Gel groups (Fig. 2f, g), which were consistent with the quantitative analysis of TAMs and CD8^+^ T cells at the tumor site (Fig. 2c, d). Collectively, the PLX-NP@Gel displayed a potent TAM depleting capability for modulating immunosuppressive tumor microenvironment, leading to the enhanced infiltration of CD8^+^ T cells for potentially improved tumor immunotherapy.

### In vivo anti-tumor efficacy of PLX-NP-P-aPD-1@Gel in tumor recurrence models of CT26 colon cancer and B16F10 melanoma

**Fig. 3:**
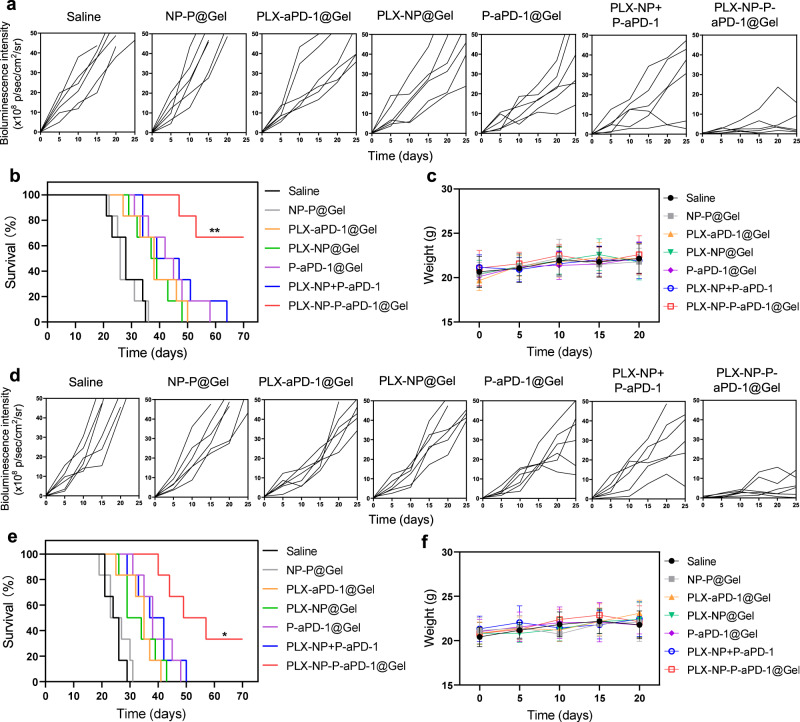
Evaluation of in vivo treatment efficacy of PLX-NP-P-aPD-1@Gel in CT26 and B16F10 tumor recurrence models. **a** Region-of-interest analysis of bioluminescence intensity of the tumors among different treatment groups in CT26 tumor recurrence model (*n* = 6 mice). Saline; NP-P@Gel, blank nanoparticle and unmodified platelets co-loaded hydrogel; PLX-aPD-1@Gel, free PLX and aPD-1 co-loaded hydrogel; PLX-NP@Gel, PLX-NP loaded hydrogel; P-aPD-1@Gel, P-aPD-1 loaded hydrogel; PLX-NP+P-aPD-1, free PLX-NP and P-aPD-1; PLX-NP-P-aPD-1@Gel, PLX-NP and P-aPD-1 co-loaded hydrogel. The doses of PLX and aPD-1 were 5 mg/kg and 0.1 mg/kg, respectively. **b** Survival curves of the mice treated with different treatment groups in CT26 tumor recurrence model (*n* = 6 mice). Data were analyzed with Log-rank (Mantel-Cox) test, PLX-NP-P-aPD-1@Gel vs. PLX-NP+P-aPD-1, ^**^P = 0.0092. **c** The weight change of mice among different treatment groups in CT26 tumor recurrence model. Data are presented as mean ± s. d. (*n* = 6 mice). **d** Region-of-interest analysis of bioluminescence intensity of the tumors among different treatment groups in B16F10 tumor recurrence model (*n* = 6 mice). **e** Survival curves of the mice treated with different treatment groups in B16F10 tumor recurrence model (*n* = 6 mice). Data were analyzed with Log-rank (Mantel-Cox) test, PLX-NP-P-aPD-1@Gel vs. PLX-NP+P-aPD-1, ^*^P = 0.0303. **f** The weight change of mice among different treatment groups in B16F10 tumor recurrence model. Data are presented as mean ± s. d. (*n* = 6 mice). Source data are provided as a Source Data file.

To further demonstrate the immunotherapeutic efficacy of PLX-NP-P-aPD-1@Gel, a CT26 colon cancer recurrence mouse model was established and treated with various treatment groups. The recurrent tumor growth was monitored by measuring bioluminescence signals from luciferase-tagged CT26 cells during the time-course of treatment, which displayed a negligible difference among all resected tumors initially (Supplementary Fig. 11). Afterwards, various treatments were applied to the postoperative mice, including saline, NP-P@Gel (blank nanoparticle and unmodified platelets co-loaded hydrogel), PLX-aPD-1@Gel (free PLX and aPD-1 co-loaded hydrogel), PLX-NP@Gel (PLX-NP loaded hydrogel), P-aPD-1@Gel (P-aPD-1 loaded hydrogel), PLX-NP+P-aPD-1 (free PLX-NP and P-aPD-1), PLX-NP-P-aPD-1@Gel (PLX-NP and P-aPD-1 co-loaded hydrogel) at the doses of 5 mg/kg PLX and 0.1 mg/kg aPD-1. Bioluminescence signals of tumors were utilized to monitor the tumor growth during the time-course of treatment. The tumor growth of mice in the NP-P@Gel group was barely inhibited compared to the saline group, where all the mice died within 36 days (Fig. 3a and Supplementary Fig. 11). Moreover, compared with moderate anti-tumor effects of the PLX-aPD-1@Gel, PLX-NP@Gel, P-aPD-1@Gel, and PLX-NP+P-aPD-1, the tumor growth was remarkably suppressed in the PLX-NP-P-aPD-1@Gel-treated mice, demonstrating the potent therapeutic efficiency of PLX-NP-P-aPD-1@Gel in this colon cancer recurrence model (Fig. 3a and Supplementary Fig. 11). Moreover, PLX-NP-P-aPD-1@Gel significantly prolonged the survival of the mice, with over 60% of the mice alive for 70 days (Fig. 3b). Furthermore, the biocompatibility of PLX-NP-P-aPD-1@Gel with no obvious toxicity was indicated by no significant weight loss of mice during the time-course of treatment (Fig. 3c).

To further evaluate the anti-tumor efficacy of PLX-NP-P-aPD-1@Gel against post-surgery tumor recurrence, a B16F10 melanoma recurrence mouse model was built, and surgery was performed to implant the hydrogel based delivery systems. As shown in Supplementary Fig. 12, after surgery, the bioluminescence intensity of tumor was barely observed, and there was no significant difference among different groups. As shown in Fig. 3d and Supplementary Fig. 12, while the NP-P@Gel group showed negligible anti-tumor effect compared to the saline group, mice treated with PLX-aPD-1@Gel, PLX-NP@Gel, P-aPD-1@Gel, PLX-NP+P-aPD-1, and PLX-NP-P-aPD-1@Gel resulted in varying efficacy in the inhibition of tumor growth, evidenced by sharper increases of bioluminescence signals in the saline and NP-P@Gel groups compared with all other groups. The implantation of PLX-NP@Gel and P-aPD-1@Gel at the surgical cavities displayed moderate tumor growth inhibition effects, indicating the limited therapeutic efficacy of localized treatment of PLX-NP or P-aPD-1 as a monotherapy strategy. While in combination therapeutic groups, mice treated with PLX-aPD-1@Gel and PLX-NP+P-aPD-1 did not display promising anti-tumor effects. However, mice treated with PLX-NP-P-aPD-1@Gel showed the most prominent protection from tumor recurrence, which substantiated the superiority of the hydrogel as a local reservoir for the sustained and bioresponsive release of PLX and aPD-1. It is worth noting that a single treatment of a low dose of P-aPD-1 (0.1 mg/kg of aPD-1) displayed a significant improvement in the inhibition of tumor recurrence when integrating with PLX-NP into the hydrogel compared to all other treatment groups, highlighting the advantages of convergence of TAM depletion and immune checkpoint blockade. The more potent anti-tumor activity of PLX-NP-P-aPD-1@Gel was further demonstrated by the prolonged survival time of the mice compared with all other groups (Fig. 3e). Moreover, no significant weight loss of mice was observed in all treatment groups during the time-course of treatment, suggesting negligible toxicities against mice (Fig. 3f).

### In vivo TAM depletion and CD8^+^ T cell infiltration after PLX-NP-P-aPD-1@Gel treatment

**Fig. 4:**
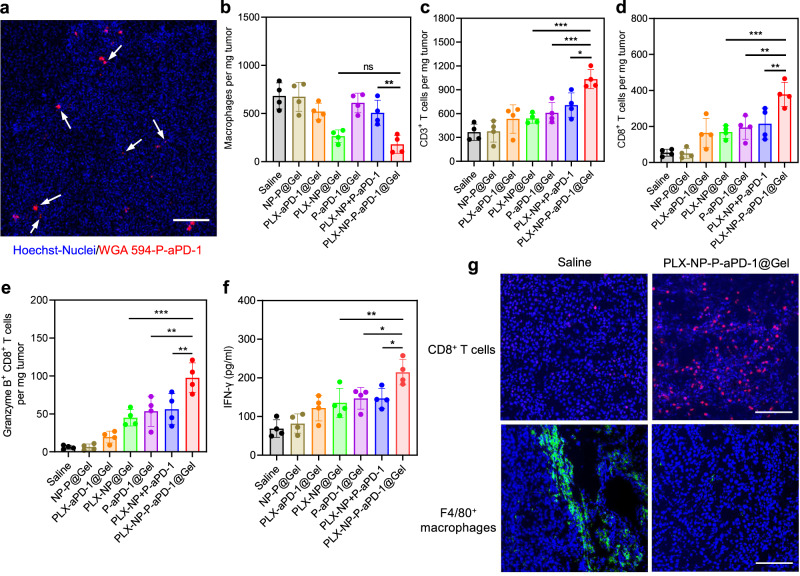
Evaluation of in vivo immune response of PLX-NP-P-aPD-1@Gel in B16F10 tumor recurrence model. **a** Representative confocal microscopy images of platelets and the formation of platelet-derived microparticles at the tumor site (*n* = 3 biologically independent samples). Scale bar, 100 µm. **b**–**e** Quantitative analysis of the number of F4/80^+^ macrophages (**b**, PLX-NP-P-aPD-1@Gel vs. PLX-NP@Gel: ^ns^*P* = 0.7842; PLX-NP-P-aPD-1@Gel vs. PLX-NP+P-aPD-1: ^**^P = 0.0026), CD3^+^ T cells (**c**, PLX-NP-P-aPD-1@Gel vs. PLX-NP@Gel: ^***^P = 0.0002; PLX-NP-P-aPD-1@Gel vs. P-aPD-1@Gel: ^***^P = 0.0009; PLX-NP-P-aPD-1@Gel vs. PLX-NP+P-aPD-1: ^*^P = 0.0100), CD8^+^ T cells (**d**, PLX-NP-P-aPD-1@Gel vs. PLX-NP@Gel: ^***^P = 0.0004; PLX-NP-P-aPD-1@Gel vs. P-aPD-1@Gel: ^**^P = 0.0015; PLX-NP-P-aPD-1@Gel vs. PLX-NP+P-aPD-1: ^**^P = 0.0049), and Granzyme B^+^ CD8^+^ T cells (**e**, PLX-NP-P-aPD-1@Gel vs. PLX-NP@Gel: ^***^P = 0.0002; PLX-NP-P-aPD-1@Gel vs. P-aPD-1@Gel: ^**^P = 0.0012; PLX-NP-P-aPD-1@Gel vs. PLX-NP+P-aPD-1: ^**^P = 0.0023) per tumor mass in the recurrent tumor tissues after treatments with saline, NP-P@Gel, PLX-aPD-1@Gel, PLX-NP@Gel, P-aPD-1@Gel, PLX-NP+P-aPD-1, and PLX-NP-P-aPD-1@Gel. Data are presented as mean ± s.d. (*n* = 4 biologically independent samples) and analyzed with one-way ANOVA followed by Dunnett’s multiple comparison test. **f** IFNγ levels in the tumors detected by enzyme-linked immunosorbent assay (ELISA) after different treatments (PLX-NP-P-aPD-1@Gel vs. PLX-NP@Gel: ^**^P = 0.0056; PLX-NP-P-aPD-1@Gel vs. P-aPD-1@Gel: ^*^P = 0.0197; PLX-NP-P-aPD-1@Gel vs. PLX-NP+P-aPD-1: ^*^P = 0.0199). Data are presented as mean ± s.d. (*n* = 4 biologically independent samples) and analyzed with one-way ANOVA followed by Dunnett’s multiple comparison test. **g** Representative confocal microscopy images of immune-stained CD8^+^ T cells and TAMs in the saline group and PLX-NP-P-aPD-1@Gel group (*n* = 3 biologically independent samples). Green: TAMs. Red: CD8^+^ T cells. Blue: Nucleus. Scale bar, 100 µm. The experiments were repeated three times. Source data are provided as a Source Data file.

To further investigate the underlying mechanism of the potent anti-tumor efficacy of PLX-NP-P-aPD-1@Gel, the ongoing activation of P-aPD-1 was first visualized in the confocal images of tumors treated with WGA 594-labeled P-aPD-1 after surgery (Fig. 4a), as evidenced by the observation of the existence of PMPs in the tumor tissues. The production of PMPs that was attributed to in situ activation of platelets could release aPD-1 antibodies in a bioresponsive manner, further re-activating exhausted CD8^+^ T cells for enhanced anti-tumor efficacy. Moreover, the activation of platelets could also help recruit CD8^+^ T cells by secreting various chemokines and cytokines, including CD40L^36^ and RANTES^37^, strengthening the effects of immune checkpoint blockade treatment. Next, after the various treatments, tumor tissues were collected for flow cytometry analysis. As shown in Fig. 4b, the number of macrophages in the tumor tissues implanted with all hydrogels containing PLX-NP formulations were decreased compared with other groups, in which PLX-NP-P-aPD-1@Gel displayed a 73.7% reduction of macrophages when compared to the saline group. There was no significant difference in the density of macrophages between the PLX-NP-P-aPD-1@Gel group and the PLX-NP@Gel group. We further analyzed T cells in the tumor tissues to investigate if there was any enhancement of infiltrated T cells after depletion of TAMs. As shown in Fig. 4c, the population of CD3^+^ T cells in the PLX-NP@Gel and P-aPD-1@gel group was increased by 1.5-fold and 1.7-fold, respectively, when compared with the saline group. Furthermore, a significantly enhanced CD3^+^ T cell infiltration was observed when co-delivering PLX-NP and P-aPD-1 in a hydrogel, as evidenced by 2.8-fold, 1.9-fold, and 1.7-fold increases in the number of CD3^+^ T cells compared with saline, PLX-NP@Gel, and P-aPD-1@Gel groups, respectively. In addition, a significantly increased number of CD8^+^ T cells in the tumor tissues treated with all aPD-1 formulations were observed compared with other treatment groups, while the highest density of CD8^+^ T cells in the tumor tissue was achieved in the PLX-NP-P-aPD-1@Gel group, which was 2-fold higher than that in the P-aPD-1@Gel group, highlighting that the TAM depletion by PLX-NP contributed substantially to the enhanced infiltration of CD8^+^ T cells (Fig. 4d). Meanwhile, a significant increase in Granzyme B^+^ CD8^+^ T cells was seen in the PLX-NP-P-aPD-1@Gel group compared with all other treatment groups, indicating an enhanced effector T cell population for stronger anti-tumor effects (Fig. 4e). Furthermore, the IFNγ cytokine level was elevated in the PLX-NP+P-aPD-1@Gel group (Fig. 4f), demonstrating the strengthened immune response. To further demonstrate the TAM depletion and improved CD8^+^ T cell infiltration, immunohistochemistry staining was performed on tumor tissues. As shown in Fig. 4g, a substantial depletion of TAMs and an increased number of infiltrated CD8^+^ T cells were observed in the PLX-NP-P-aPD-1@Gel group than those in the saline group. Additionally, the hematoxylin and eosin (H&E) staining of the main organs of the mice showed therapeutic safety of the PLX-NP-P-aPD-1@Gel treatment strategy as there was negligible evidence of systemic toxicity compared to saline-treated mice (Supplementary Fig. 13). Collectively, the results revealed that the TAM depletion by PLX-NP released from the hydrogel facilitated the infiltration of CD8^+^ T cells by modulating the tumor immunosuppressive microenvironment, which was further activated by aPD-1 antibodies released from P-aPD-1, enhancing immunotherapeutic efficacy.

### In vivo anti-tumor efficacy of the PLX-NP-P-aPD-1@Gel in 4T1 breast tumor recurrence and metastasis models

**Fig. 5:**
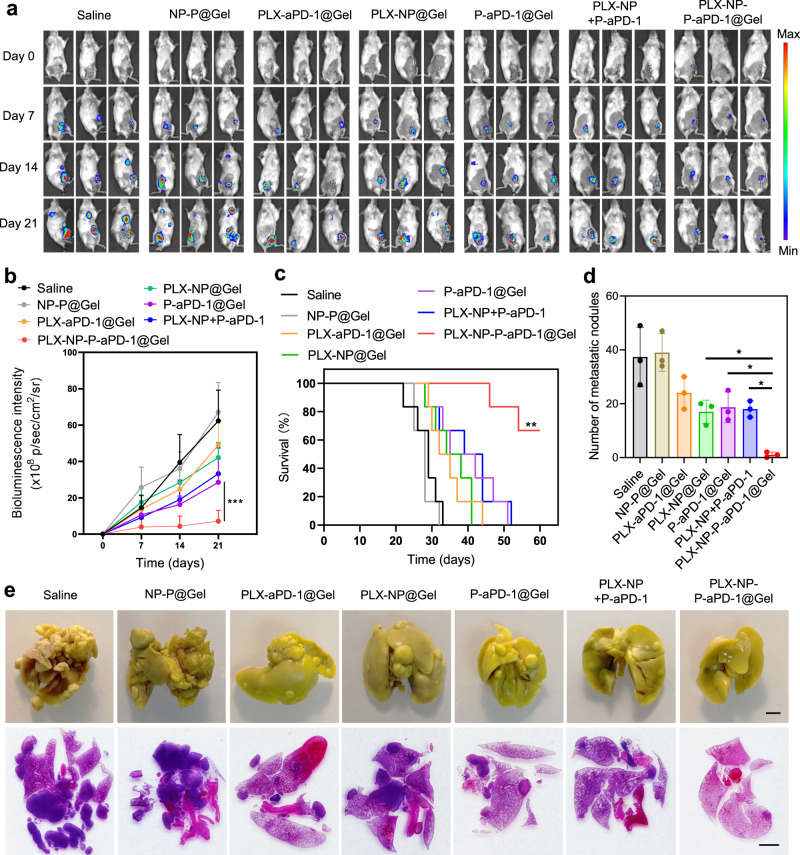
Evaluation of in vivo treatment efficacy of PLX-NP-P-aPD-1@Gel in metastatic 4T1 breast tumor recurrence model. **a** Representative bioluminescence images of 4T1 tumor-bearing mice following different treatments on day 0, day 7, day 14, day 21 (*n* = 6 mice). Saline; NP-P@Gel, blank nanoparticles and unmodified platelets co-loaded hydrogel; PLX-aPD-1@Gel, free PLX and aPD-1 co-loaded hydrogel; PLX-NP@Gel, PLX-NP loaded hydrogel; P-aPD-1@Gel, P-aPD-1 loaded hydrogel; PLX-NP+P-aPD-1, free PLX-NP and P-aPD-1; PLX-NP-P-aPD-1@Gel, PLX-NP and P-aPD-1 co-loaded hydrogel. The dose of PLX and aPD-1 was 5 mg/kg and 0.1 mg/kg, respectively. **b** Region-of-interest analysis of bioluminescence intensity of the tumors among different treatment groups (PLX-NP-P-aPD-1@Gel vs. PLX-NP+P-aPD-1: ^***^P < 0.0001). Data are presented as mean ± s.d. (*n* = 6 mice) and analyzed with two-way ANOVA followed by Tukey’s multiple comparisons test. **c** Survival curves of the mice treated with different treatment groups (*n* = 6 mice). Data were analyzed with Log-rank (Mantel-Cox) test, PLX-NP-P-aPD-1@Gel vs. PLX-NP + P-aPD-1: ^**^P = 0.0018. **d** Quantitative analysis of the number of metastatic nodules on the surface of the lungs on day 21 (PLX-NP-P-aPD-1@Gel vs. PLX-NP@Gel: ^*^P = 0.0313; *P*LX-NP-P-aPD-1@Gel vs. P-aPD-1@Gel: ^*^P = 0.0168; PLX-NP-P-aPD-1@Gel vs. PLX-NP+P-aPD-1: ^*^P = 0.0215). Data are presented as mean ± s.d. (*n* = 3 biologically independent samples) and analyzed with one-way ANOVA followed by Dunnett’s multiple comparison test. **e** Representative images of the lungs after fixation in bouin’s solution and H&E staining after different treatments on day 21 (*n* = 3 biological independent samples, scale bar = 2 mm). Source data are provided as a Source Data file.

Clinically, triple-negative breast cancer is highly aggressive and metastatic, which usually does not respond effectively to the immune checkpoint blockade therapy^38,39^. Therefore, to further demonstrate the immunotherapeutic efficacy of PLX-NP-P-aPD-1@Gel in a breast tumor model, the 4T1 breast tumor recurrence model was established and treated with various treatment groups. The recurrent tumor growth was monitored by measuring bioluminescence signals from luciferase-tagged 4T1 cells during the time-course of treatment. As shown in Fig. 5a, the surgical bed displayed negligible bioluminescence signals on day 0 after surgery. Not surprisingly, as shown in Fig. 5a, the bioluminescence signal in the saline group began to spread to the lung area on day 14, and became enormously strong on day 21, indicating that the tumor had recurred from the surgical site and metastasized to the lung. The NP-P@Gel showed almost no therapeutic effect for either the tumor recurrence or lung metastasis. Notably, the PLX-NP-P-aPD-1@Gel, potently inhibited the tumor recurrence and further growth, showing significantly better anti-tumor efficacy than the PLX-aPD-1@Gel, PLX-NP@Gel, P-aPD-1@Gel, and PLX-NP+P-aPD-1 treatment groups (Fig. 5b). Moreover, the mice in the saline group all died within 33 days, while after the treatment of PLX-NP+P-aPD-1, more than 66% of mice survived more than 60 days, which is superior to other treatment strategies (Fig. 5c). In addition, we established the 4T1 breast tumor recurrence-induced lung metastasis model and performed corresponding treatments to further explore the effects of our local treatment strategy for the inhibition of lung metastasis. On day 21, all the mice were sacrificed, and the lungs were collected for analysis. We found that the PLX-NP-P-aPD-1@Gel treatment could significantly decrease the number of metastatic nodules on the lung surface compared with other treatment groups (Fig. 5d). Also, as visualized in Fig. 5e, we observed severe lung metastasis in the saline group and found that the PLX-aPD-1@Gel, PLX-NP@Gel, P-aPD-1@Gel, and PLX-NP+P-aPD-1 treatments can inhibit lung metastasis to a certain extent. Encouragingly, the PLX-NP-P-aPD-1@Gel exhibited the most potent inhibition of lung metastasis.

### In vivo anti-tumor efficacy of PLX-NP-P-aPD-1@Gel in the recurrent S180 sarcoma model, B16F10 tumor recurrence model in Rag^-/-^ mice and B16F10 distant tumor model

**Fig. 6:**
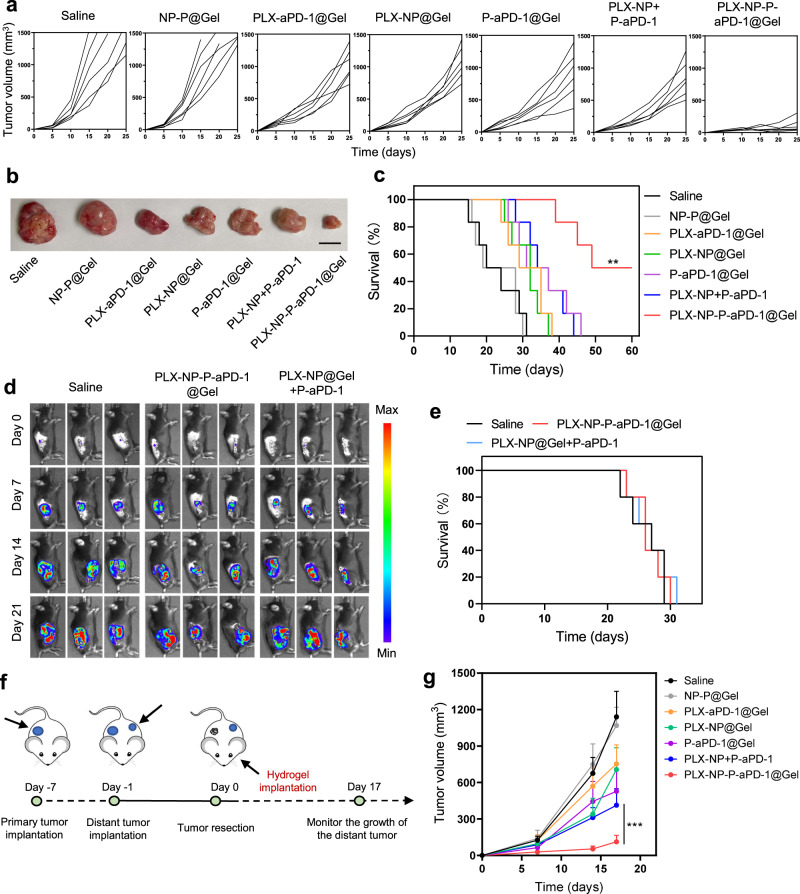
Evaluation of in vivo treatment efficacy of PLX-NP-P-aPD-1@Gel in different tumor recurrence models including the sarcoma S180 tumor model and B16F10 melanoma model in T cell-deficient mice. **a** Tumor growth curves among different treatment groups in sarcoma S180 tumor recurrence model (*n* = 6 mice). Saline; NP-P@Gel, blank nanoparticles and unmodified platelets co-loaded hydrogel; PLX-aPD-1@Gel, free PLX and aPD-1 co-loaded hydrogel; PLX-NP@Gel, PLX-NP loaded hydrogel; P-aPD-1@Gel, P-aPD-1 loaded hydrogel; PLX-NP+P-aPD-1, free PLX-NP and P-aPD-1; PLX-NP-P-aPD-1@Gel, PLX-NP and P-aPD-1 co-loaded hydrogel. The doses of PLX and aPD-1 were 5 mg/kg and 0.1 mg/kg, respectively. **b** The images of representative tumors after different treatments on day 21 (*n* = 6 mice). **c** Survival curves of the mice in different treatment groups in the S180 tumor recurrence model (*n* = 6 mice). Data were analyzed with Log-rank (Mantel-Cox) test, PLX-NP-P-aPD-1@Gel vs. PLX-NP+P-aPD-1: ^**^P = 0.0031. **d** Representative bioluminescence images of B16F10 tumor-bearing Rag^−/−^ mice following different treatments on day 0, day 7, day 14, day 21 (*n* = 5 mice). **e** Survival curves of the mice treated with different treatment groups in B16F10 tumor recurrence model in Rag^−/−^ mice (*n* = 5 mice). **f** Schematic illustration of the establishment and treatment strategy of a double tumor model. **g** Tumor growth curve of the distant tumor after different treatments (PLX-NP-P-aPD-1@Gel vs. PLX-NP+P-aPD-1: ^***^P <  0.0001). Data are presented as mean ± s.d. (*n* = 6 mice) and analyzed with two-way ANOVA followed by Tukey’s multiple comparison test. Source data are provided as a Source Data file.

Here, we further established an S180 sarcoma tumor model, a notorious tumor with aggressiveness and limited immunotherapeutic response. As shown in Fig. 6a, b, the tumors in the saline group recurred and grew quickly, and tumor-bearing mice all died within 31 days. Although the PLX-aPD-1@Gel, PLX-NP@Gel, P-aPD-1@Gel, and PLX-NP+P-aPD-1 treatments showed moderate inhibitions of tumor recurrence and growth, all the mice still died within 46 days. Notably, the PLX-NP-P-aPD-1@Gel treatment strategy significantly inhibited the growth of the recurred tumor, and 50% of the mice survived to 60 days (Fig. 6c). Furthermore, to demonstrate the importance of T cells in our hydrogel-based post-surgery immunotherapy, we established the recurring B16F10 melanoma model in T cell-deficient Rag^−/−^ mice and evaluated the anti-tumor efficacy of PLX-NP-P-aPD-1@Gel and PLX-NP@Gel+P-aPD-1 by measuring the bioluminescence signals from luciferase-tagged B16F10 cells. As shown in Fig. 6d, e, there were no significant differences in anti-tumor effects between either treatment groups or the saline control group. Moreover, the tumor-bearing Rag^−/−^ mice in different treatment groups all died within 31 days (Fig. 6e), further validating that T cells play a fundamental role in the enhanced immune response of PLX-NP-P-aPD-1@Gel. In order to evaluate if our local treatment strategy could activate a systemic immune response able to inhibit the growth of a distant tumor, we established a B16F10 distant tumor model. As shown in Fig. 6f, we established a primary B16F10 tumor on one side of the mouse and injected B16F10 tumor cells on the other side after six days. On day 0, we resected the primary tumor and implanted the hydrogel delivery systems into the surgical bed, and the volumes of distant tumors were monitored (Fig. 6f). As shown in Fig. 6g, compared with other treatments, the growth of the distant tumor in the PLX-NP-P-aPD-1@Gel was significantly inhibited, demonstrating that our local treatment strategy could activate a systemic immune response to inhibit distant disease.

### In vivo anti-tumor efficacy of local implantation of PLX-NP@Gel and systemic injection of P-aPD-1

**Fig. 7:**
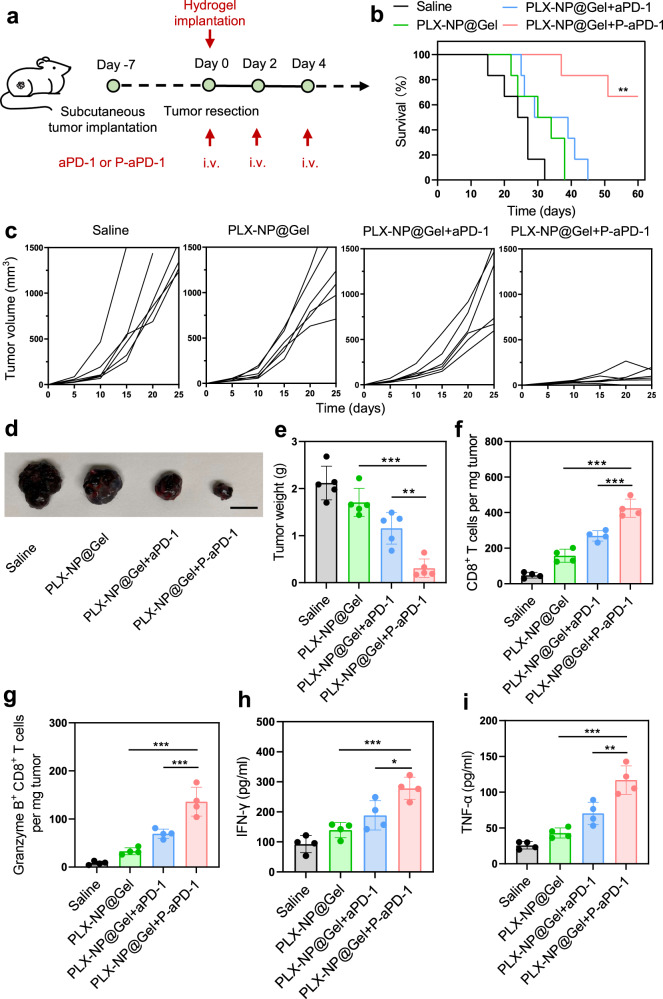
Evaluation of in vivo treatment efficacy of local implantation of PLX-NP@Gel with intravenous injection of P-aPD-1 in B16F10 tumor recurrence model. **a** Schematic illustration of the experimental design of local implantation of PLX-NP@Gel with intravenous injection of P-aPD-1 in B16F10 tumor recurrence model. **b** Survival curves of the mice treated with different groups (*n* = 6 mice). Saline; PLX-NP@Gel; PLX-NP@Gel+aPD-1, PLX-NP@Gel and intravenous injection of free aPD-1; PLX-NP@Gel+P-aPD-1, PLX-NP@Gel and intravenous injection of P-aPD-1. The dose of PLX was 5 mg/kg, and the dose of aPD-1 was 0.5 mg/kg. Data were analyzed with Log-rank (Mantel-Cox) test, PLX-NP@Gel+P-aPD-1 vs. PLX-NP@Gel+aPD-1: ^**^P = 0.0049. **c** Tumor volume change of the mice treated with different groups in three weeks (*n* = 6 mice). **d** Representative tumor images of mice among different groups on day 21 (*n* = 5 mice). Scale bar, 1 cm. **e** Tumor weight of mice among different groups on day 21 (PLX-NP@Gel+P-aPD-1 vs. PLX-NP@Gel: ^***^P < 0.0001; PLX-NP@Gel+P-aPD-1 vs. PLX-NP@Gel+aPD-1: ^**^P =  0.0012). Data are presented as mean ± s.d. (*n* = 5 mice) and analyzed with one-way ANOVA followed by Dunnett’s multiple comparisons test. **f**, **g** Quantitative analysis of the flow cytometry results of CD8^+^ T cells (**f**, PLX-NP@Gel+P-aPD-1 vs. PLX-NP@Gel: ^***^P < 0.0001; PLX-NP@Gel+P-aPD-1 vs. PLX-NP@Gel+aPD-1: ^***^P = 0.0001) and Granzyme B^+^ CD8^+^ T cells (**g**, PLX-NP@Gel+P-aPD-1 vs. PLX-NP@Gel: ^***^P < 0.0001; PLX-NP@Gel+P-aPD-1 vs. PLX-NP@Gel+aPD-1: ^***^P = 0.0003) in the recurrent tumor tissues after different treatments. Data are presented as mean ± s.d. (*n* = 4 biologically independent samples) and analyzed with one-way ANOVA followed by Dunnett’s multiple comparisons test. **h**, **i** IFNγ (**h**, PLX-NP@Gel+P-aPD-1 vs. PLX-NP@Gel: ^***^P = 0.0004; PLX-NP@Gel+P-aPD-1 vs. PLX-NP@Gel+aPD-1: ^*^P = 0.0107) and TNFα (**i**, PLX-NP@Gel+P-aPD-1 vs. PLX-NP@Gel: ^***^P < 0.0001; PLX-NP@Gel+P-aPD-1 vs. PLX-NP@Gel+aPD-1: ^**^P = 0.0010) levels within the tumor were detected by ELISA among different groups. Data are presented as mean ± s.d. (*n* = 4 biologically independent samples) and analyzed with one-way ANOVA followed by Dunnett’s multiple comparison test. Source data are provided as a Source Data file.

To investigate whether localized implantation of PLX-NP@Gel could enhance the anti-tumor effect of the systemic injection of P-aPD-1, the therapeutic efficacy of PLX-NP@Gel and systemic administration of P-aPD-1 (designated PLX-NP@Gel+P-aPD-1) was evaluated in the B16F10 tumor recurrence model. After tumor resection, different treatments were applied, including saline, PLX-NP@Gel, PLX-NP@Gel with systemic injection of free aPD-1 antibodies (PLX-NP@Gel+aPD-1), and PLX-NP@Gel+P-aPD-1 (Fig. 7a). Notably, the mice in PLX-NP@Gel+P-aPD-1 group showed markedly prolonged survival time compared with the mice in other groups (Fig. 7b), while the mice treated with saline all died within 32 days. Furthermore, as shown in Fig. 7c, PLX-NP@Gel and PLX-NP@Gel+aPD-1 treatments moderately slowed down the growth of recurrent tumors in the mice compared with the saline group while eventually failing to inhibit the tumor growth. In contrast, the tumor recurrence and growth were significantly prevented by PLX-NP@Gel+P-aPD-1 treatment. The enhanced tumor inhibition of PLX-NP@Gel+P-aPD-1 compared to PLX-NP@Gel+aPD-1 highlighted the critical role of platelets in the in vivo delivery of immune checkpoint inhibitors, which could improve (1) pharmacokinetics of aPD-1 antibodies by prolonging the circulation time^28^; (2) accumulation of aPD-1 antibodies at the tumor site by leveraging the homing capability of platelets towards the wound site and the tumor tissue^40,41^. Furthermore, as visualized in Fig. 7d, mice treated with PLX-NP@Gel+P-aPD-1 carried the smallest tumors with the lowest tumor weight among all treatment groups (Fig. 7e). In addition, we directly compared the treatment efficacy of local gel-released P-aPD-1 (single dose) to systemic P-aPD-1 (single dose and 3 doses). As shown in supplementary Fig. S14, we found that the therapeutic efficacy of local gel-released P-aPD-1 (0.1 mg/kg of aPD-1, single dose) was better than that of the systemic P-aPD-1 treatment (0.5 mg/kg of aPD-1, single dose), which could be attributed to the insufficient accumulation of P-aPD-1 at the tumor site at a relatively low dose (0.5 mg/kg of aPD-1) after systemic administration to trigger a robust immune response. However, three dosages of systemic P-aPD-1 treatment showed the best tumor inhibition and treatment efficacy compared to both single dose of local gel-release P-aPD-1 and systemic P-aPD-1. Moreover, the immune response was further investigated by flow cytometry and the ELISA assay of cytokines after PLX-NP@Gel+P-aPD-1 treatment. Enhanced CD8^+^ T cell infiltration in PLX-NP@Gel and PLX-NP@Gel+aPD-1 groups were quantitatively demonstrated with 3.3-fold and 5.7-fold increases compared with the saline group, respectively (Fig. 7f). In contrast, the PLX-NP@Gel+P-aPD-1 group showed a 1.6-fold greater number of CD8^+^ T cells compared with the PLX-NP@Gel+aPD-1 group, which could be attributed to the increased pharmacokinetics and tumor-selective accumulation of aPD-1 mediated by platelets. Moreover, the promotion of T cell activation was substantiated by increased Granzyme B^+^ CD8^+^ T cell population in mice treated with PLX-NP@Gel+P-aPD-1 compared with all other groups (Fig. 7g). Furthermore, elevated cytokine levels were detected in the PLX-NP@Gel+P-aPD-1 group, as shown by a 3-fold increase in IFN*γ* (Fig. 7h) and a 4.5-fold increase in TNF*α* (Fig. 7i) compared with the saline group. Taken together, this local TAM depletion strategy facilitated both local and systemic treatment efficacy of platelet-mediated delivery of the aPD-1 immune checkpoint inhibitor.

## Discussion

We have shown here that the hydrogel could act as a local reservoir to provide sustained releases of PLX-NP and P-aPD-1 for the enhanced efficacy of tumor immunotherapy by depleting TAMs to facilitate T cell infiltration and in situ promoting aPD-1 release in a bioresponsive manner for blocking the PD-1/PD-L1 pathway to re-activate infiltrated T cells. Furthermore, this local TAM depletion strategy could be further adapted to enhance the treatment outcomes of systemic platelet-mediated aPD-1 delivery.

Inhibition of TAMs by blocking CSF1R is a viable method to modulate tumor immunosuppressive microenvironment to facilitate CD8^+^ T cells infiltration, while the durable treatment outcomes are yet to be achieved in the clinic partially due to the nonspecific distribution of CSF1R inhibitors that could also deplete macrophages in healthy tissues, leading to side effects like edema^42–44^. Here we propose a local delivery strategy to embed PLX-NP into a hydrogel implanted in the post-surgery tumor cavity. The hydrogel can act as a depot for controlled and sustained release of PLX concentrated in the tumor tissue against TAMs, which will minimize the side effects toward normal tissues and augment the depletion efficacy of PLX. Furthermore, previous studies have demonstrated that TAMs could impede the infiltration of CD8^+^ T cells, limiting the treatment efficacy of immune checkpoint blockade strategy^42^. Encouragingly, depletion of TAMs could facilitate the migration of CD8^+^ T cells towards tumor parenchyma by blocking the crosstalk between CD8^+^ T cells and TAMs, promoting anti-tumor immune response. However, the delivery of immune checkpoint inhibitors, especially when administrated systemically, often suffers from a quick clearance, diminishing their therapeutic efficacy^45,46^. In this study, we employed platelets as carriers for aPD-1 antibodies and embedded them into the hydrogel together with PLX-NP. The sustained release of P-aPD-1 could be controlled by the hydrogel, followed by the presentation of aPD-1 towards T cells facilitated by in situ activation of platelets in the inflammatory environment of the post-surgical tumor site^4,47,48^. Furthermore, the tissue damage associated with the surgical incisions would also induce platelet adherence and localization, thereby concentrating the P-aPD-1 to the area near the tumor, rather than wide-systemic distribution as seen with intravenous administration of checkpoint inhibiting monoclonal antibodies. Additionally, the platelet activation in the inflammatory environment secondary to the tumor surgery could also facilitate the recruitment of immune cells, boosting the anti-tumor immune response^49,50^.

We further demonstrate that this local depletion of TAMs through the hydrogel reservoir could also augment the immunotherapy efficacy of systemic injection of P-aPD-1, diversifying the administration routes of immune checkpoint inhibitors. To be noted, the systemic free aPD-1 injection did not cause a significant improvement in therapeutic outcomes here, which could be attributed to the low availability of aPD-1 antibodies at the tumor site. While the enhanced treatment efficacy in the combination of systemic P-aPD-1 and PLX-NP@Gel suggested the key role of platelets in promoting the systemic biodistribution of intravenously injected aPD-1 by selectively accumulating at the tumor site, subsequently synergizing with TAM depletion-mediated modulation of the tumor immunosuppressive environment for improved efficacy^50,51^.

For future translatability of PLX-NP-P-aPD-1@Gel, the materials (dextran and alginate) applied to prepare nanoparticles and hydrogel have already been approved by the U.S. Food and Drug Administration (FDA) for medical uses. Furthermore, the application of endogenous platelets as delivery carriers for aPD-1 antibodies will minimize the immunogenicity of drug delivery systems and alleviate the quick clearance that is challenging for most synthetic delivery systems in the clinic. To outlook the future translation of PLX-NP-P-aPD-1@Gel, the PLX-NP-loaded hydrogel could be lyophilized and easily stocked and serve as an off-the-shelf drug formulation to be readily applied to the surgical cavity post-operation after loading with freshly prepared P-aPD-1 to prevent cancer recurrence. For P-aPD-1, how to ensure the manufacturing quality of engineering platelets will be critical for its future translation. Furthermore, since platelets are quite sensitive to environmental changes, more advanced storage of P-aPD-1 will limit its wide application in the clinic and more friendly storage and transportation methods will facilitate further clinical applications.

Collectively, we have demonstrated that PLX-NP and P-aPD-1 could be delivered as a combination treatment through an alginate-based hydrogel localized tumoral delivery after surgical resection, facilitating the treatment efficacy by leveraging the synergy of TAM depletion and bioresponsive aPD-1 delivery. Furthermore, the local TAM elimination approach could also improve the treatment outcomes of systemic P-aPD-1 delivery. Moreover, this strategy could be adapted to facilitate other immunotherapeutic modalities whose efficacy has been hindered significantly by the tumor immunosuppressive environment, such as adoptive T cell therapy^52–54^ and cancer vaccination^55,56^.

## Methods

### Antibodies and cells

The mouse melanoma B16F10 cells, CT26 cells and 4T1 cells were tagged with luciferase for in vivo bioluminescence imaging. The B16F10, NIH/3T3, Sarcoma 180 (S180) and Raw 264.7 cells were purchased from ATCC. Luciferase-expressed B16F10 cells, CT26 cells and 4T1 cells were obtained from Imanis Life Sciences Inc. Cells were cultured in the CO_2_ incubator (Fisher) at 37 °C with 5% CO_2_ and 90% relative humidity. The cells were sub-cultured about every 2 days at 80% confluence. The antibodies used in this study were summarized as follows (company, clone, category number): GoInVivo Purified anti-mouse CD279 (PD-1) (BioLegend, RMP1-14, 114114), Fluorescein isothiocyanate (FITC)-anti-mouse CD45 (BioLegend, 30-F11, 103108), APC-anti-mouse F4/80 (BioLegend, BM8, 123116), PerCP/Cy5.5-anti-human/mouse CD11b (BioLegend, M1/70, 101227), APC-anti-mouse CD3 (BioLegend, 17A2, 100236), FITC-anti-mouse CD4 (BioLegend, GK1.5, 100406), PE-anti-mouse CD8a (BioLegend, 53-6.7, 100708), FITC-anti-mouse IFN*γ* (BioLegend, XMG1.2, 505806), PerCP/Cy5.5-anti-human/mouse Granzyme B (BioLegend, QA16A02, 372212), PE-anti-mouse CD45 (BioLegend, 30-F11, 103106), FITC-anti-human/mouse CD11b (BioLegend, M1/70, 101206), Alexa Fluro 594 anti-mouse CD8a (BioLegend, 53-6.7, 100758), Alexa Fluro 647 anti-mouse F4/80 (BioLegend, BM8, 123121), PE-anti-mouse CD62P (BioLegend, RMP-1, 124807), PE-anti-mouse CD41 (BioLegend, MWReg30, 133905), FITC-anti-mouse CD9 (BioLegend, MZ3, 148305), FITC-anti-mouse CD61 (BioLegend, 2C9.G2, 104305), Precision Count Beads (BioLegend, 424902). All antibody dilutions were performed following the manufacture’s guidance (diluted ~200 times for immediate use). FlowJo software was used to analyze flow cytometry data.

### Preparation and characterization of PLX-NP

Dextran was modified with Pyridinium P-toluenesulfonate (PPTS) and 2-ethoxypropene for the preparation of nanoparticles. Briefly, 1 g dextran was dissolved in 10 ml anhydrous dimethyl sulfoxide, and 0.062 mmol PPTS (Sigma Aldrich) and 37 mmol 2-ethoxypropene (Matrix Scientific) were added to the dextran solution during stirring. After 30 min, the reaction was quenched by adding 1 ml triethylamine (Sigma Aldrich) during stirring at room temperature, resulting in 2-ethoxypropene-modified dextran (designated m-dextran). m-dextran was then precipitated in the basic water and collected by centrifugation.

To prepare PLX-NP, 10 mg m-dextran and 0.5 mg PLX were firstly dissolved in 2 ml dichloromethane (DCM). Afterwards, 4 ml 3% (w/v) poly (vinyl alcohol) (PVA) solution was then slowly added to the DCM solution followed by the sonication for emulsification. Then, the emulsion was added to 20 ml 0.3% (w/v) PVA solution and stirred for one hour for solvent evaporation. The nanoparticles were collected by centrifugation at 21,900 g for 45 min. The resulted PLX-NP was analyzed by DLS to determine the average size, and the morphology of nanoparticles was characterized by TEM. To study the in vitro release property of PLX, PLX-NP was suspended in 3 ml phosphate-buffered saline (PBS, pH 6.5) with 0.1% Tween 80 and loaded into a 3 ml 20,000 MWCO dialysis cassette (Thermo scientific). The cassette was placed into a container with 4 L PBS with 0.1% v/v Tween 80, and at predetermined time points, 10 µl supernatant was collected, dissolved by acetonitrile, and the concentration was analyzed by high-performance liquid chromatography (HPLC). Similarly, for the in vivo drug release of PLX at the tumor site, the surgical removal of tumor mass and the implantation of the hydrogel systems into the mice were performed. And then, at different time points, the hydrogel systems were taken out and de-crosslinked with EDTA, and the remaining drug concentration was measured by HPLC as described above. To further test the cytotoxicity of PLX-NP against macrophages and normal cells, the cell viability of Raw 264.7 and NIH/3T3 cells treated with different concentrations (0 to 125 µg/ml) of PLX-NP for 24 h was determined by MTT assay.

### Preparation and characterization of anti-PD-1-conjugated platelets

The mouse platelets were purified from whole mouse blood collected by retro-orbital bleeding. Afterwards, the blood was centrifuged for 20 min at 100 g, followed by centrifugation for 20 min at 800 g. The platelets were collected and suspended in PBS with the addition of 1 µM PGE1 to prevent platelet activation. To conjugate aPD-1 antibodies on the surface of platelets, aPD-1 antibodies were first reacted with SMCC linkers for 2 h at 4 °C at a molar ratio of 1:1.2. And then, the excess SMCC linkers were discarded by centrifugation at 21,900 g for 10 min at 4 °C, using 3000 KDa MWCO ultrafiltration tubes. The synthesized SMCC-aPD-1 was added into Traut’s reagent-pretreated platelets and stirred at room temperature for 1 h to obtain P-aPD-1. The excess antibodies were removed by centrifugation at 1500 g for 20 min. To characterize the conjugation efficacy of aPD-1 with platelets, P-aPD-1 was subjected to 0.1% Triton-X100 buffer to release aPD-1 under unltrasonication, and the amount of aPD-1 was determined by ELISA kit (Rat IgG total ELISA Kit, Invitrogen).

To demonstrate the successful conjugation of aPD-1 on the surface of platelets, confocal microscopy (Nikon A1RS) imaging and flow cytometry (ThermoFisher Attune) analysis were performed. Briefly, aPD-1 was stained by FITC, and platelets were stained by Wheat Germ Agglutinin 594 (WGA 594). And then, the functionality of aPD-1-conjugated platelets was studied by two assays: collagen binding assay and surface antigen expression study. First, collagen from the human placenta (Sigma) was reconstituted to a concentration of 1.0 mg/ml and was added to a confocal dish for incubation overnight at 4 °C. The coated confocal dishes and uncoated confocal dishes were further blocked with 1 ml 2% (w/v) bovine serum albumin in PBS for two hours and washed with PBS. Rhodamine B-labeled naïve platelets and P-aPD-1 were then added to the dishes and incubated for 5 min. The unbinding platelets and P-aPD-1 were washed with PBS, and then the dishes were visualized under the confocal microscope and analyzed with NIS-Elements viewer. The surface protein expression of P-aPD-1 was investigated by flow cytometry by staining with various antibodies (CD61, CD41, CD9 diluted by ~200 times), compared with unmodified platelets. Furthermore, the platelet activation marker CD62P was characterized by flow cytometry after P-aPD-1 was treated with thrombin.

### Preparation of alginate-based hydrogel

The alginate-based hydrogel was formed by adding 10 µl 100 mg/ml CaCl_2_ solution into 200 µl 10 mg/ml alginate solution in HEPES buffered saline. To study the in vivo degradation rate of alginate-based hydrogel, alginate was conjugated with Cy5.5. Briefly, 50 mg sodium alginate was dissolved in 5 ml of HEPES buffer (50 mM, pH = 5), 14 mg NHS, 116 mg EDC, and 18 mg NH_2_-PEG_3_-N_3_, and the mixture were stirred for 30 min at the room temperature. Then the pH of the solution was adjusted to 7.5–8.0 and reacted overnight at the room temperature. The synthesized alginate-N_3_ was purified by a 3-day dialysis against water. The 100 µl of 1% (w/v) solution of alginate-N_3_ was incubated with 30 µl of 1 mM Cy5.5-DBCO for four hours. The final product was purified using dialysis against water. The synthesized alginate-Cy5.5 was mixed with unreacted alginate at a volume ratio of 1:1 to form Cy5.5-labeled hydrogel. The Cy5.5-labeled hydrogel was implanted into the C57BL/6 mice subcutaneously, and the fluorescence signals were monitored by IVIS (Perkin Elmer).

### Characterization of hydrogel loaded with PLX-NP and P-aPD-1

The predetermined amounts of PLX-NP and P-aPD-1 were suspended into 50 µl HEPES buffered saline and mixed with 10 µl 100 mg/ml CaCl_2_ solution, adding to 200 µl 10 mg/ml alginate solution in HEPES buffered saline to form PLX-NP-P-aPD-1@Gel. The Cryo SEM imaging was first performed to visualize the morphology of PLX-NP-P-aPD-1@Gel, and confocal microscopy was also used to characterize the successful loading of NP and P-aPD-1 in the hydrogel. WGA 594 labeled P-aPD-1 and FITC-loaded NP were prepared and loaded into the alginate hydrogel, and the hydrogel was then observed under the confocal microscope.

To examine the loading capacity of alginate-based hydrogel, different volumes of suspension containing a fixed amount of PLX-NP and P-aPD-1 were loaded into a 200 µl alginate solution in a 96-well plate. After 3 min, hydrogels were removed from wells, and the P-aPD-1 was counted using hemocytometers under the microscope, and the remaining amounts of PLX were determined by HPLC.

To study the platelet and aPD-1 release from hydrogel, hydrogel containing 1 × 10^8^ P-aPD-1 was placed into a 40 µm cell strainer in a 6-well plate and submerged by 5 ml medium. Afterwards, 1 U/ml thrombin was added to trigger the activation of platelets. At predetermined time points, 50 µl samples were collected, and the same amount of medium was added back to the wells. The platelets in collected samples were counted using hemocytometers under the microscope. And then, the collected samples were centrifuged for 20 min at 800 g, and the concentration of aPD-1 in the supernatant was detected by rat total IgG ELISA kit.

### In vivo macrophage depletion ability of PLX-NP@Gel

The C57BL/6 mice (Male, aged 6–8 weeks) were purchased from Jackson laboratory. The animal study protocol was approved by the Institutional Animal Care and Use Committee (IACUC) at the University of Wisconsin-Madison. To build the in vivo mouse B16F10 melanoma recurrence model, B16F10 cells were injected into the right flank of C57BL/6 mice subcutaneously. Once the tumor size reached about 150 mm^3^, tumors were surgically removed as much as possible under a microscope, and different treatments were then applied to the tumor cavities, including saline, NP@Gel, free PLX, PLX-NP, and PLX-NP@Gel. The free PLX and PLX-NP were slowly dripped onto the tumor cavity after surgery. Tumors were collected on day 12 for following studies. Collected tumor tissues were digested by collagenase and then were dissociated by tissue dissociator (gentalMACS) to obtain single-cell suspension. The cell suspension was stained with FITC-anti-mouse CD45, APC-anti-mouse F4/80, APC-anti-mouse CD3, PerCP/Cy5.5-anti-human/mouse CD11b, FITC-anti-mouse CD4, PE-anti-mouse CD8a, FITC-anti-mouse IFN*γ* antibodies and analyzed using flow cytometry with Attune NxT Flow Cytometer software (All these antibodies were diluted by ~200 times). Furthermore, the collected tumors were embedded in optimal cutting temperature (OCT) compound and frozen in a −80 °C freezer for sectioning. To directly visualize the macrophages and T cells, the section slides were stained by the Alexa Fluro 594 anti-mouse CD8a, Alexa Fluro 647 anti-mouse F4/80 antibodies, and Hoechst 33342 trihydrochloride (Invitrogen, H3570), and then were imaged by the confocal microscope.

### In vivo anti-tumor efficacy of PLX-NP-P-aPD-1@Gel

The in vivo mouse B16F10 melanoma recurrence model was established as described before. The in vivo mouse CT26 colon cancer recurrence model was established by subcutaneously injecting CT26 cells in the right flank of the BALB/c mouse. The in vivo mouse 4T1 breast cancer recurrence model was established by injecting 4T1 cells into the BALB/c mouse mammary fat pad. Both B16F10, CT26 and 4T1 cells were tagged with luciferase for in vivo bioluminescence imaging. For the above tumor models, different treatments were applied to the tumor cavities, including saline, NP-P@Gel (blank nanoparticle and unmodified platelets co-loaded hydrogel), PLX-aPD-1@Gel (free PLX and aPD-1 co-loaded hydrogel), PLX-NP@Gel (PLX-NP loaded hydrogel), P-aPD-1@Gel (P-aPD-1 loaded hydrogel), PLX-NP+P-aPD-1 (free PLX-NP and P-aPD-1), PLX-NP-P-aPD-1@Gel (PLX-NP and P-aPD-1 co-loaded hydrogel). The free PLX and PLX-NP were slowly dripped onto the tumor cavity after surgery. The dose of aPD-1 was 0.1 mg/kg, and the dose of PLX was 5 mg/kg. From day 0, at predetermined days, the bioluminescence signals of the resected tumor tissues were monitored by IVIS, after intraperitoneally injecting 150 mg/kg D-luciferin per mice in 100 μl PBS. Mice were imaged after 5 min with 0.5-second exposure. Bioluminescence images were analyzed using Living Image Software v.4.3.1 (Perkin Elmer). The weight and survival of mice were monitored during the time-course of treatment. Once the tumor volume was larger than 1.5 cm^3^ (calculated based on the equation: length × width^2^ × 0.5), mice were euthanized following the animal protocols. We further established a 4T1 metastasis model to investigate whether PLX-NP-P-aPD-1@Gel could prevent lung metastasis after local implantation. The 4T1 local recurrence model was built and treated with the hydrogel-based delivery systems. On day 21, the lungs of the mice in different treatment groups were harvested and fixed with Bouin’s solution. The lung metastasis in saline treatment group was validated as evidenced by visible metastatic nodules and further H&E staining. After the lung tissue was photographed, the number of tumor metastases on the surface was counted. H&E assay was performed to observe the tumor metastases inside the lungs. Also, the S180 sarcoma tumor recurrence model was established and treated with the similar method as mentioned above. The recurrent tumor volume and survival of the mice were monitored accordingly. The in vivo mouse B16F10 melanoma recurrence model in T deficient mice (Rag^−/−^ mice) was established and the tumor growth and survival were monitored accordingly. To evaluate the treatment efficacy of PLX-NP-P-aPD-1@Gel to the distant tumor, we first established a primary B16F10 tumor model in the right flank of the mice and 6 days later, the distant tumor model was established by injecting B16F10 tumor cells into the left flank of the mice. Then one day later, the primary tumor was resected, and the hydrogel-based treatment systems were implanted. The growth and tumor volume of the distant tumor were monitored accordingly.

To investigate whether P-aPD-1 could be activated at the tumor site, WGA 594-labeled P-aPD-1 was loaded into the hydrogel. One week after subcutaneous B16F10 tumor implantation, the tumor was surgically resected with ~5% tumor mass left, and hydrogel was put into the tumor cavity beside the residue tumor. After three days, the residue tumor was collected for the frozen section. The section slides were imaged under the confocal microscope.

To study in vivo macrophage depletion ability and enhanced T cell infiltration of NP-PLX-P-aPD-1@Gel, the resection tumor model was established as above-mentioned and treated with saline, NP-P@Gel, PLX-aPD-1@Gel, PLX-NP@Gel, P-aPD-1@Gel, PLX-NP+P-aPD-1, PLX-NP-P-aPD-1@Gel. The free PLX and PLX-NP were slowly dripped onto the tumor cavity after surgery. On day 12, tumors were collected and digested by collagenase and then were dissociated by a tissue dissociator to obtain single-cell suspension. The cell suspension was stained with PE-anti-mouse CD45, FITC-anti-human/mouse CD11b, APC-anti-mouse F4/80, APC-anti-mouse CD3, FITC-anti-mouse CD4, PE-anti-mouse CD8a, and PerCP/Cy5.5-anti-human/mouse Granzyme B antibodies, and analyzed using flow cytometry (All these antibodies were diluted by ~200 times). Furthermore, the collected tumors were also embedded in OCT for frozen section. The section slides were stained by the Alexa Fluro 594 anti-mouse CD8a, Alexa Fluro 647 anti-mouse F4/80 antibodies, and Hoechst 33342 trihydrochloride for observation under the confocal microscope (All these antibodies were diluted by ~200 times). Moreover, to study the cytokine generation after treatments, tumor tissues were collected after 12-day treatments. The tumor tissues were resuspended in NP40 Cell Lysis Buffer (Alfa Aesar) at 4 °C and then were mechanically grounded. The homogenate was centrifuged for 10 min at 4,000 g, at 4 °C to collect the supernatant. Afterwards, 10 µl supernatant was used to be detected using corresponding cytokine ELISA kits following the manufacture’s guidance.

### In vivo anti-tumor efficacy of PLX-NP@Gel and systemic injection of P-aPD-1

To build the in vivo mouse B16F10 melanoma recurrence model, B16F10 cells were injected into the right flank of C57BL/6 mice subcutaneously. Once the tumor size reached about 150 mm^3^, surgery was performed to remove tumor mass as much as possible under a microscope, and different treatments were applied to the tumor cavities, including saline, PLX-NP@Gel, PLX-NP@Gel with intravenous injection of free aPD-1 antibodies every other day for three times starting from day 0, and PLX-NP@Gel with intravenous injection of P-aPD-1 every other day for three times starting from day 0. The dose for aPD-1 was 0.5 mg/kg per mice, and the dose for PLX was 5 mg/kg per mice. From day 0, the volumes of the tumor tissues were measured using a digital caliper and calculated based on the equation: length × width^2^ × 0.5. The survival of mice was recorded for 60 days. The maximal tumor size permitted by our animal protocol approved by the IACUC at the University of Wisconsin-Madison is 2 cm^3^. Once the tumor volume is larger than 1.5 cm^3^, mice will be euthanized following the animal protocols to prevent further suffering of the mice in this study. Moreover, the tumor tissues were collected for weight measure at three weeks after the hydrogel implantation, and the representative tumor tissues from each group were imaged. To study in vivo macrophage depletion ability and enhanced T cell infiltration of different groups, the flow cytometry, and the ELISA assays for IFNγ and TNF*α* were performed as mentioned above.

### Statistics

All the results are shown as mean ± s.d. The GraphPad Prism software was used to perform statistical analysis and ANOVA was used to compare multiple groups (>two groups) statistically. Log-rank test was performed for the statistical analysis of the survival study. A *P* value lower than 0.05 (*P < 0.05) was considered as the threshold for statistical significance among control groups and experimental groups.

### Reporting summary

Further information on research design is available in the Nature Research Reporting Summary linked to this article.

## Supplementary information

Supplementary Information

Reporting Summary

## Data Availability

The authors declare that all the data supporting the findings of this study are available within the article, Supplementary Information and Source Data file. Source data are provided with this paper.

## Source data

Source Data
