# Supplementary material for: Depletion of tumor associated macrophages enhances local and systemic platelet-mediated anti-PD-1 delivery for post-surgery tumor recurrence treatment
Source: Nat Commun. 2022 Apr 6;13:1845. doi: 10.1038/s41467-022-29388-0 (PMC8987059; doi:10.1038/s41467-022-29388-0)
Supplement: Supplementary file 1 — Supplementary Information [file 41467_2022_29388_MOESM1_ESM.pdf]

Supplementary Information

Depletion of Tumor Associated Macrophages Enhances Local and Systemic Platelet-Mediated Anti-PD-1 Delivery for Post-Surgery Tumor Recurrence Treatment

Zhaoting Li[1,2,3#], Yingyue Ding[1,2,3#], Jun Liu[1,2,3], Jianxin Wang[1,3], Fanyi Mo[1,2,3], Yixin Wang[1,2,3], Ting-Jing Chen-Mayfield[1,2,3], Paul M. Sondel[2,4,5], Seungpyo Hong[1,2,3], Quanyin Hu[1,2,3,*]

[1]Pharmaceutical Sciences Division, School of Pharmacy, University of Wisconsin-Madison, Madison, WI 53705, United States

[2]Carbone Cancer Center, School of Medicine and Public Health, University of Wisconsin-Madison, Madison, WI 53705, United States

[3]Wisconsin Center for NanoBioSystems, School of Pharmacy, University of Wisconsin-Madison, Madison, WI 53705, United States

[4]Department of Human Oncology, University of Wisconsin School of Medicine and Public Health, Madison, WI, United States

[5]Department of Pediatrics, University of Wisconsin School of Medicine and Public Health, Madison, WI, United States

Corresponding Author

*Quanyin Hu, Email: qhu66@wisc.edu

[#]These authors contributed equally.

Contents

Supplementary Fig. 1. The average size and TEM image of PLX-NP.

Supplementary Fig. 2. The cell viability of RAW264.7 cell line and NIH/3T3 cell line treated with PLX-NP.

Supplementary Fig. 3. The collagen-binding evaluation of free platelets and P-aPD-1.

Supplementary Fig. 4. The flow cytometry histograms of surface protein expression of platelets and P-aPD-1.

Supplementary Fig. 5. The morphologies of the alginate-based hydrogel.

Supplementary Fig. 6. The loading efficiency of PLX-NP and platelets in the alginate hydrogel.

Supplementary Fig. 7. Representative Cryo-SEM images of the PLX-NP and P-aPD-1 co-loaded hydrogel at different magnifications.

Supplementary Fig. 8. The release profile of PLX-NP@Gel *in vivo*.

Supplementary Fig. 9. Gating strategy for flow cytometry analysis of macrophages and T lymphocytes.

Supplementary Fig.10. Representative flow cytometry assay for IFNγ staining.

Supplementary Fig. 11. Representative bioluminescence images of CT26-Luc tumor-bearing mice following different treatments.

Supplementary Fig. 12. Representative bioluminescence images of B16F10-Luc tumor-bearing mice following different treatments.

Supplementary Fig. 13. The representative H&E staining images of the heart, liver, spleen, lung, and kidney in saline and PLX-NP-P-aPD-1@Gel treatment groups.

Supplementary Fig. 14. Tumor growth curve after systemic and local treatments.

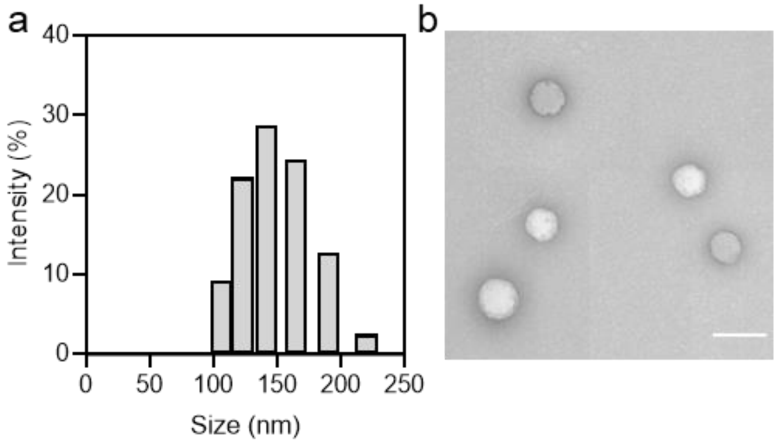

Supplementary Fig. 1. (a) The average size of PLX-NP measured by DLS. (b) The TEM image of PLX-NP. Scale bar, 200 nm. The experiments were repeated three times.

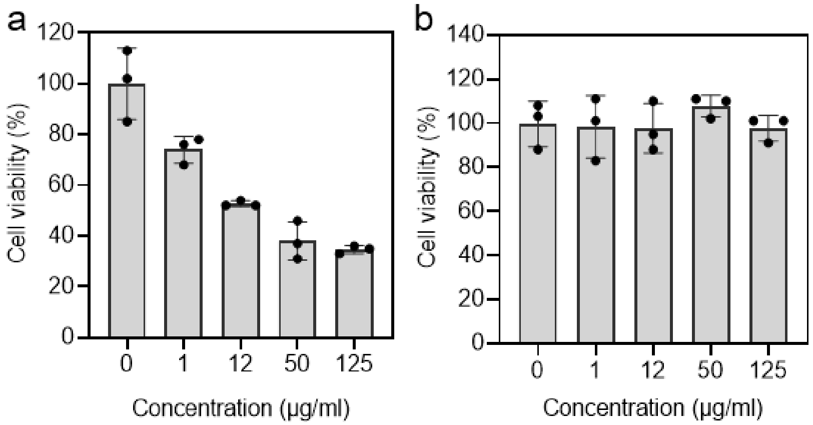

Supplementary Fig. 2. The cell viability of (a) RAW264.7 cell line and (b) NIH/3T3 cell line treated with PLX-NP. Cells were treated with various concentrations of PLX-NP ranging from 0-125 μg/ml for 24 hours. Data are presented as mean ± s.d. (n = 3).

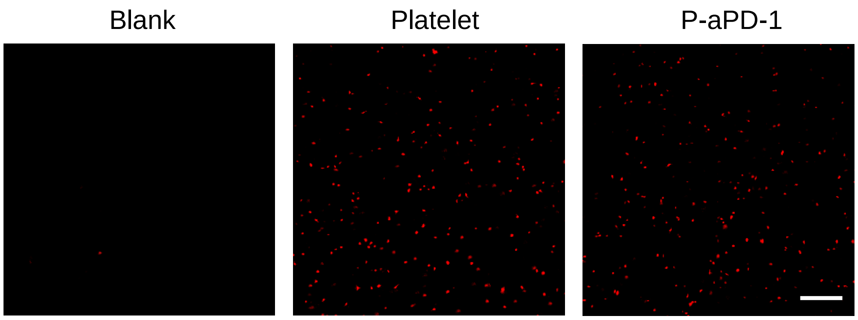

Supplementary Fig. 3. The collagen-binding evaluation of free platelets and P-aPD-1. The confocal dish without pre-treatment of collagen was set as blank control. Platelets and P-aPD-1 were labeled with NHS-Rhodamine B for confocal microscopy. Scale bar, 50 μm. The experiments were repeated three times.

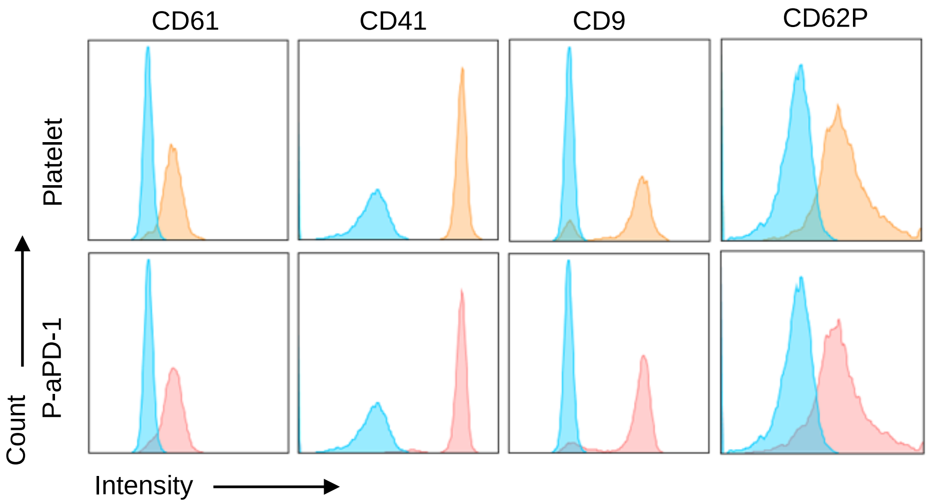

Supplementary Fig. 4. The flow cytometry histograms of surface protein expression of platelets and P-aPD-1 (n = 3). Anti-mouse CD8 antibody was selected as the isotype control antibody as shown identical in both Platelet group and P-aPD-1 group (indicated as blue color). CD62P was the platelet activation marker.

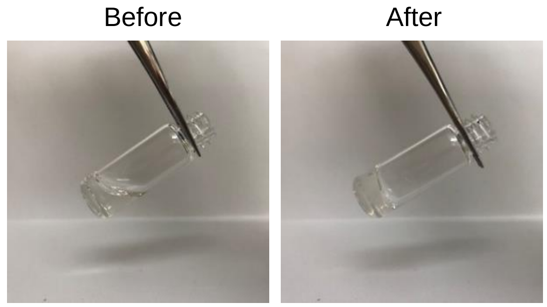

Supplementary Fig. 5. The morphologies of alginate-based hydrogel before and after adding Ca^{2+}.

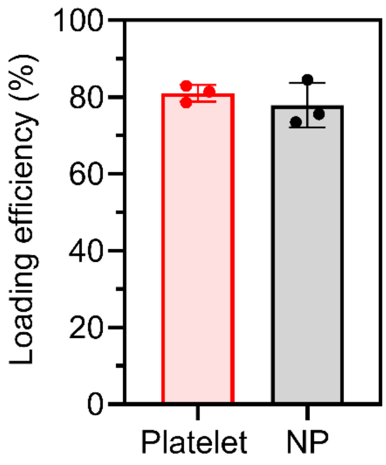

Supplementary Fig. 6. The loading efficiency of PLX-NP and platelet in the alginate hydrogel. Data are presented as mean ± s.d. (n = 3).

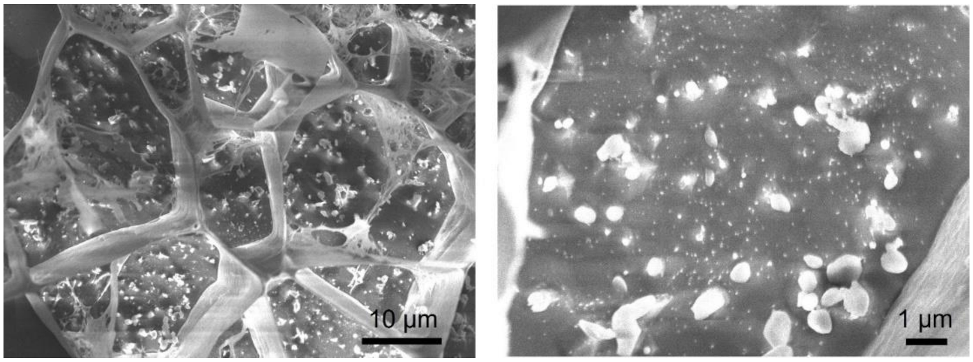

Supplementary Fig. 7. Representative Cryo-SEM images of the PLX-NP and P-aPD-1 co-loaded hydrogel at different magnifications. The experiments were repeated three times.

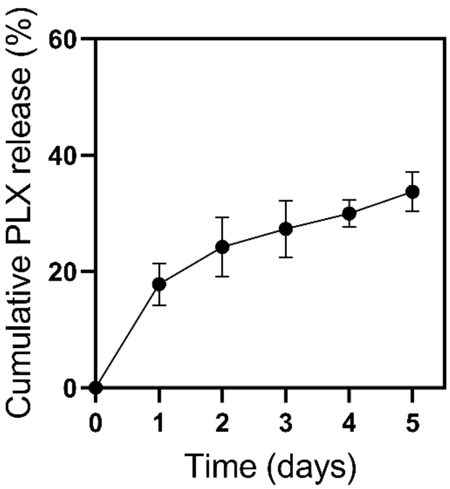

Supplementary Fig. 8. Release profile of PLX-NP@Gel *in vivo*. Data are presented as mean ± s.d. (n = 3).

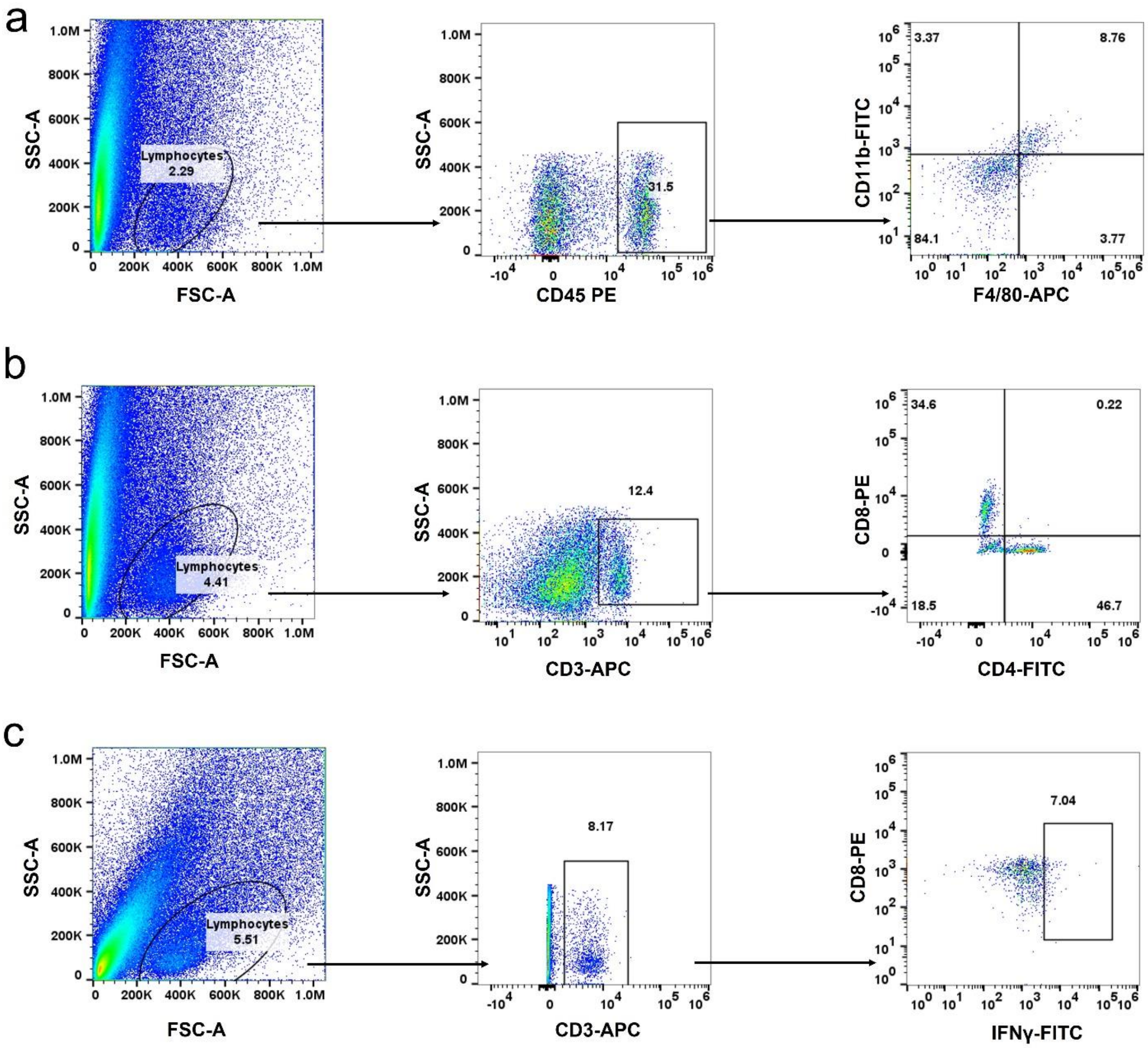

Supplementary Fig. 9. Gating strategies for flow cytometry analysis of macrophages and T lymphocytes. **a** Gating strategy for the $CD45^+F4/80^+CD11b^+$ macrophage analysis in Fig. 2a, 2c and Fig. 4b. **b** Gating strategy for the $CD3^+CD4^+CD8^+$ T lymphocytes in Fig. 2b, 2d, Fig. 4c, 4d and Fig. 7f. **c** Gating strategy for the $CD3^+CD8^+IFN\gamma^+$ T lymphocytes in Fig. 2e.

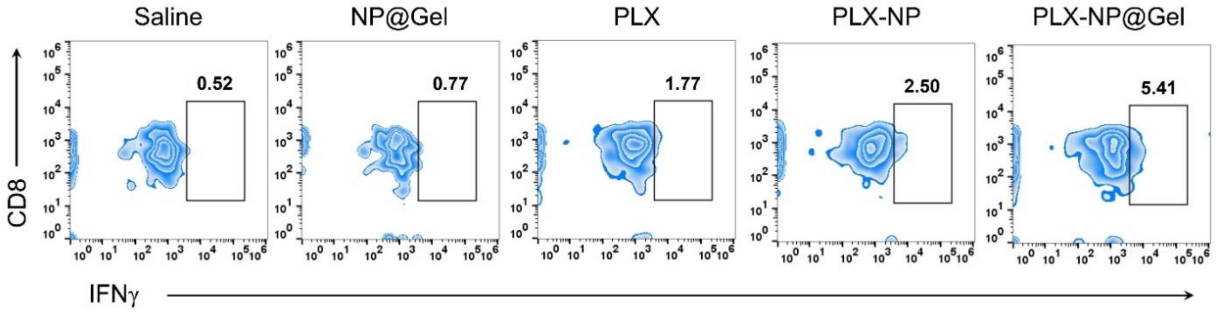

Supplementary Fig. 10. Representative flow cytometry assay for IFNγ$^+$ CD8$^+$ lymphocytes (n = 5 biologically independent samples).

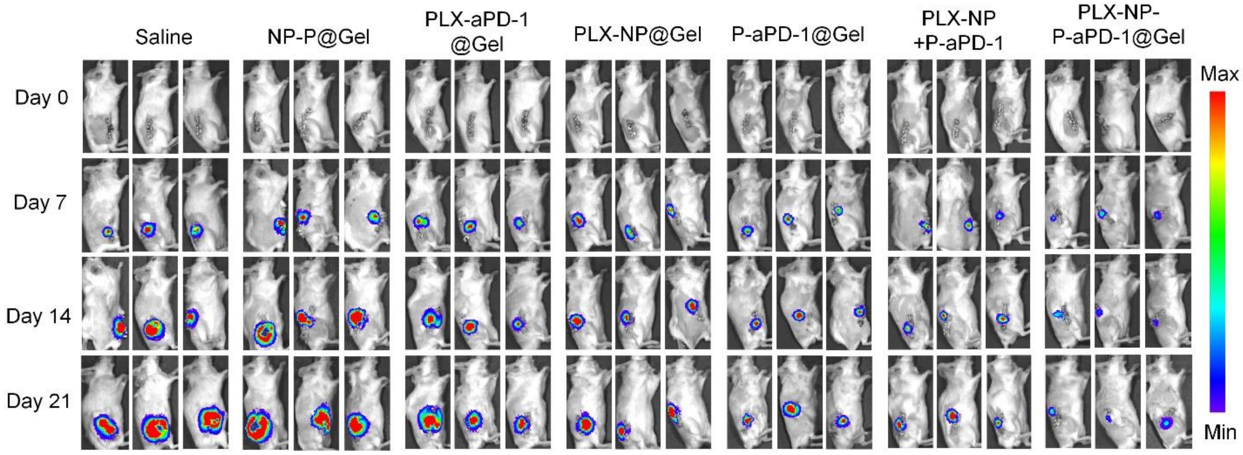

Supplementary Fig. 11. Representative bioluminescence images of CT26-Luc recurrent tumor-bearing mice following different treatments on day 0, day 7, day 14, and day 21 (n = 6 mice). Saline; NP-P@Gel, blank nanoparticle and unmodified platelets co-loaded hydrogel; PLX-aPD-1@Gel, free PLX and aPD-1 co-loaded hydrogel; PLX-NP@Gel, PLX-NP loaded hydrogel; P-aPD-1@Gel, P-aPD-1 loaded hydrogel; PLX-NP+P-aPD-1, free PLX-NP and P-aPD-1; PLX-NP-P-aPD-1@Gel, PLX-NP and P-aPD-1 co-loaded hydrogel. The doses of PLX and aPD-1 were 5 mg/kg and 0.1 mg/kg, respectively.

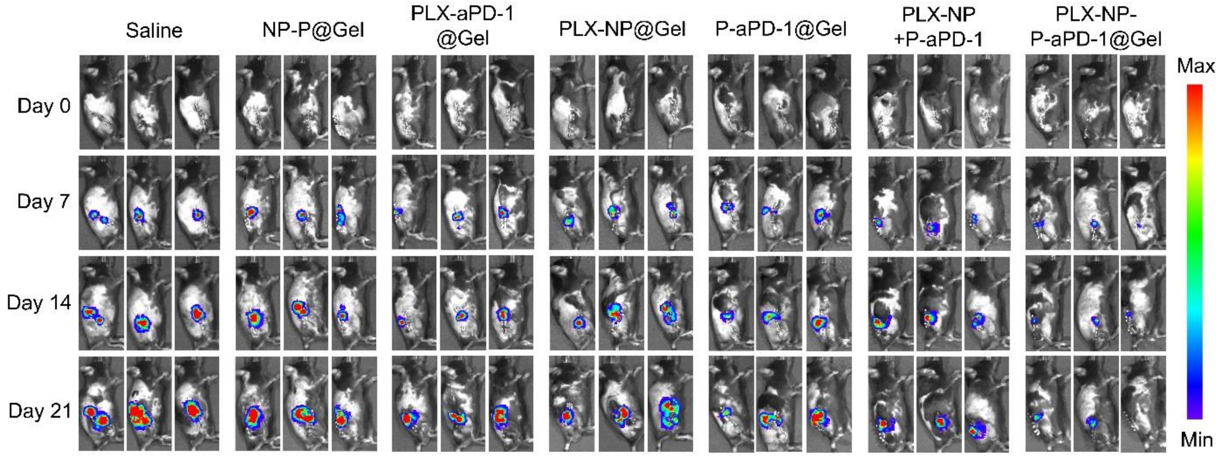

Supplementary Fig. 12. Representative bioluminescence images of B16F10-Luc recurrent tumor-bearing mice following different treatments on day 0, day 7, day 14, and day 21 (n = 6 mice). Saline; NP-P@Gel, blank nanoparticle and unmodified platelets co-loaded hydrogel; PLX-aPD-1@Gel, free PLX and aPD-1 co-loaded hydrogel; PLX-NP@Gel, PLX-NP loaded hydrogel; P-aPD-1@Gel, P-aPD-1 loaded hydrogel; PLX-NP+P-aPD-1, free PLX-NP and P-aPD-1; PLX-NP-P-aPD-1@Gel, PLX-NP and P-aPD-1 co-loaded hydrogel. The doses of PLX and aPD-1 were 5 mg/kg and 0.1 mg/kg, respectively.

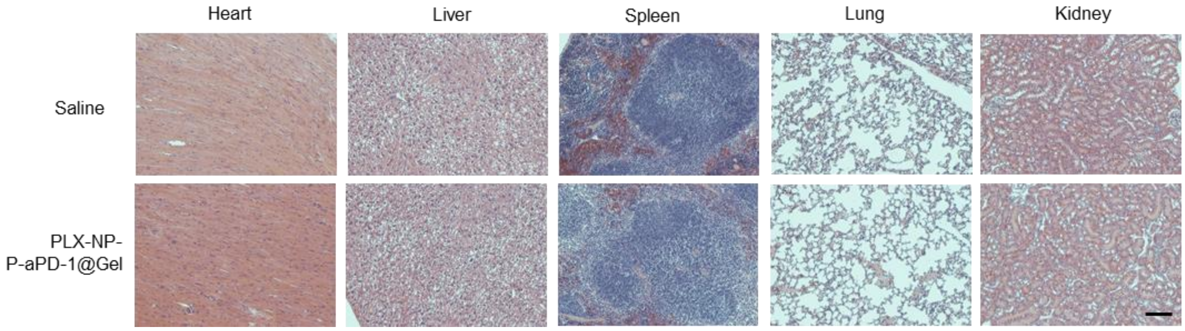

Supplementary Fig. 13. The representative H&E staining images of the heart, liver, spleen, lung, and kidney in saline and PLX-NP-P-aPD-1@Gel treatment groups (n = 3 biologically independent samples). Scale bar is 100 μm. The experiments were repeated three times.

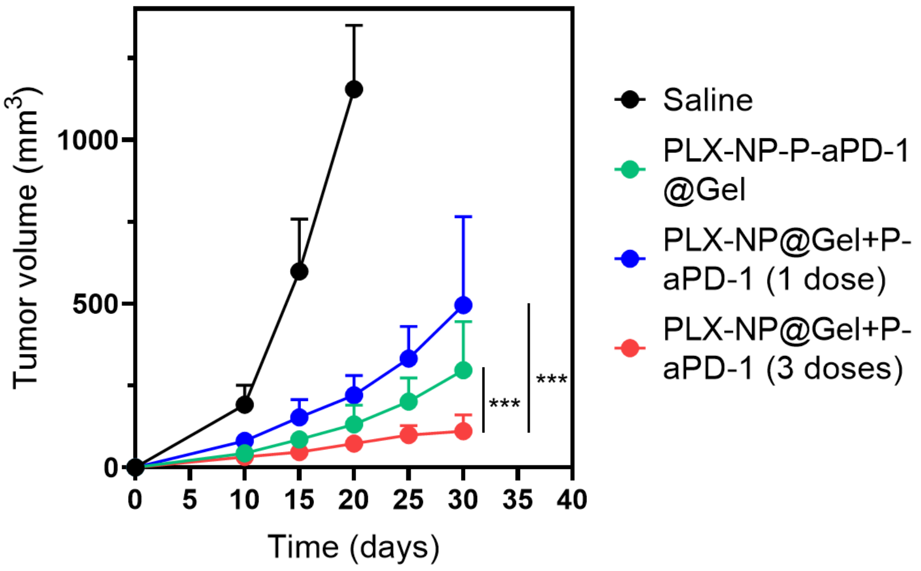

Supplementary Fig. 14. Tumor growth curve after systemic and local treatments. The dose of PLX was 5 mg/kg, the dose of aPD-1 for the PLX-NP-P-aPD-1@Gel was 0.1 mg/kg, and the dose of aPD-1 for the PLX-NP@Gel+P-aPD-1 was 0.5 mg/kg. Data are presented as mean ± s.d. (n = 6 mice) and analyzed with two-way ANOVA followed by Tukey's multiple comparison test. PLX-NP@Gel+P-aPD-1 (3 doses) vs. PLX-NP@Gel+P-aPD-1 (1 dose): $^{***}P < 0.0001$; PLX-NP@Gel+P-aPD-1 (3 doses) vs. PLX-NP-P-aPD-1@Gel: $^{***}P = 0.0006$.
